# Combatting cellular immortality in cancers by targeting the shelterin protein complex

**DOI:** 10.1186/s13062-024-00552-4

**Published:** 2024-11-22

**Authors:** Sohini Chakraborty, Satarupa Banerjee

**Affiliations:** Department of Biotechnology, School of Bioscience and Technology, Vellore Institute of Technology, Vellore, 632014 India

**Keywords:** Shelterin proteins, RNAs, Network pharmacology, Expression, Patient survival, Correlation

## Abstract

Shelterin proteins (TERF1, TERF2, TPP1, TINF2, POT1) protect telomeres, prevent unwarranted repair activation, and regulate telomerase activity. Alterations in these proteins can lead to cancer progression. This study uses an in-silico approach to examine shelterin in tumour samples across various cancers, employing mutation plots, phylogenetic trees, and sequence alignments. Network pharmacology identified TERF1 as an essential shelterin protein and transcription factors RUNX1, CTCF, and KDM2B as potential biomarkers due to their interactions with miRNAs and shelterin proteins. We performed MCODE analysis to identify subnetworks of ncRNAs interacting with the shelterin proteins. Shelterin expression predicted patient survival in 24 cancer types, with TERF1, TERF2, TINF2, and POT1 significantly expressed in testicular, AML, prostate, breast and renal cancers, respectively, and TPP1 in AML and skin cancer. Spearman and Pearson's analyses showed significant correlations of TERF1 across cancers, with near-significant correlations for all five proteins in different cancer datasets like breast cancer, kidney renal papillary and lung squamous cell carcinoma, skin cutaneous melanoma, etc.,. Shelterin expression correlated with patient survival in breast, renal, lung, skin, uterine, and gastric cancers. Insights into TPP1-associated glycans highlighted glycosylated sites contributing to tumorigenesis. This study provides molecular signatures for further functional and therapeutic research on shelterin, highlighting its potential as a target for anti-cancer therapies and promising prospects for cancer prognosis and prediction.

**Supplementary Information:**

The online version contains supplementary material available at 10.1186/s13062-024-00552-4.

## Introduction

**Fig. 1:**
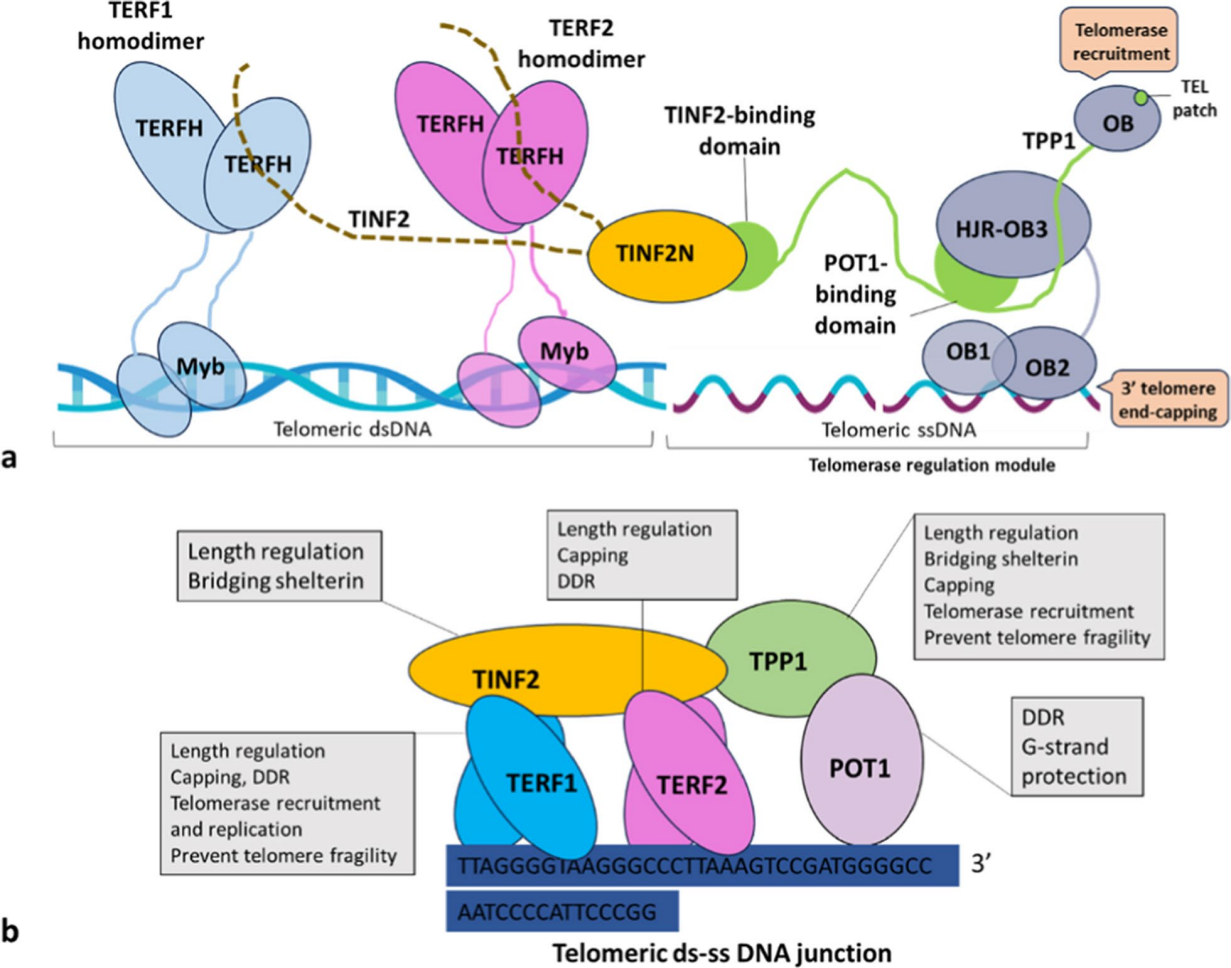
The components of the shelterin complex, telomerase and the specific functions of each shelterin component are highlighted in grey insets. Telomeric DNA is made up of both double-stranded and single-stranded DNA, and in vertebrates. This duality enables the single-stranded 3′ tail to enter the double-stranded DNA region, forming a displacement loop (D-loop) and a telomere loop (T-loop). Shelterin complexes regulate T-loop development, which can prevent telomerase from accessing the 3′ tail. Once the telomere is opened, presumably during the S phase of the cell cycle, telomerase can connect to the 3′ tail using its RNA template and add telomeric repeats. CST then blocks telomerase activity, preventing excessive telomere lengthening

Cells that undergo malignant transformation face the challenge of evading replicative senescence and eventual cell death due to progressive telomere shortening that occurs with each cell division. Telomeres are protected by a specialized protein complex called shelterin or the telosome. This complex comprises proteins TERF1, TERF2, TPP1, POT1 and TINF2, which work together to prevent telomere degradation or fusion. Shelterin plays a critical role in facilitating the formation of T-loop structures, recruiting telomerase, and protecting telomeres [1]. Moreover, the molecular components of telomerase act as additional factors that influence telomeres' replicative and protective functions. Often, these components work together with shelterin or other core telomere-binding proteins to orchestrate the intricate events that govern telomere maintenance and stability. When telomere protection is lost, it can lead to chromosome end-to-end fusions and a state of telomere crisis, characterized by extensive genome instability, chromothripsis, kataegis, and tetraploidization, all of which can promote cancer progression. Beyond its role in end-protection, shelterin also functions as a mechanism for sensing telomere length and regulates telomerase activity at the telomere. TERF1 and TERF2 are homodimers that bind to telomeric double-stranded DNA, while the POT1–TPP1 heterodimer binds and caps the telomeric 3′ tail. TINF2 connects the TERF1 and TERF2 dimers with POT1–TPP1, forming the complete shelterin complex (Fig. 1a) [2]. Aberrant shelterin expression has been observed in various cancers like breast cancer [3], adrenal cortical cancer [4], colorectal cancer [5], gastric cancer [6–8], glioblastoma [9–11], lung cancer [12], prostate cancer [13, 14], chronic lymphocytic leukaemia [15–17] and melanoma [18] with mutations in some shelterin genes acting as cancer drivers. Paradoxically, cancer cells (CCs) require adequate shelterin to sustain their rapid proliferation, and increased levels of shelterin proteins such as TERF1, TERF2, and TINF2 are associated with tumour development. This complex relationship between shelterin expression and tumorigenesis necessitates further investigation (Fig. 1).

**Fig. 2:**
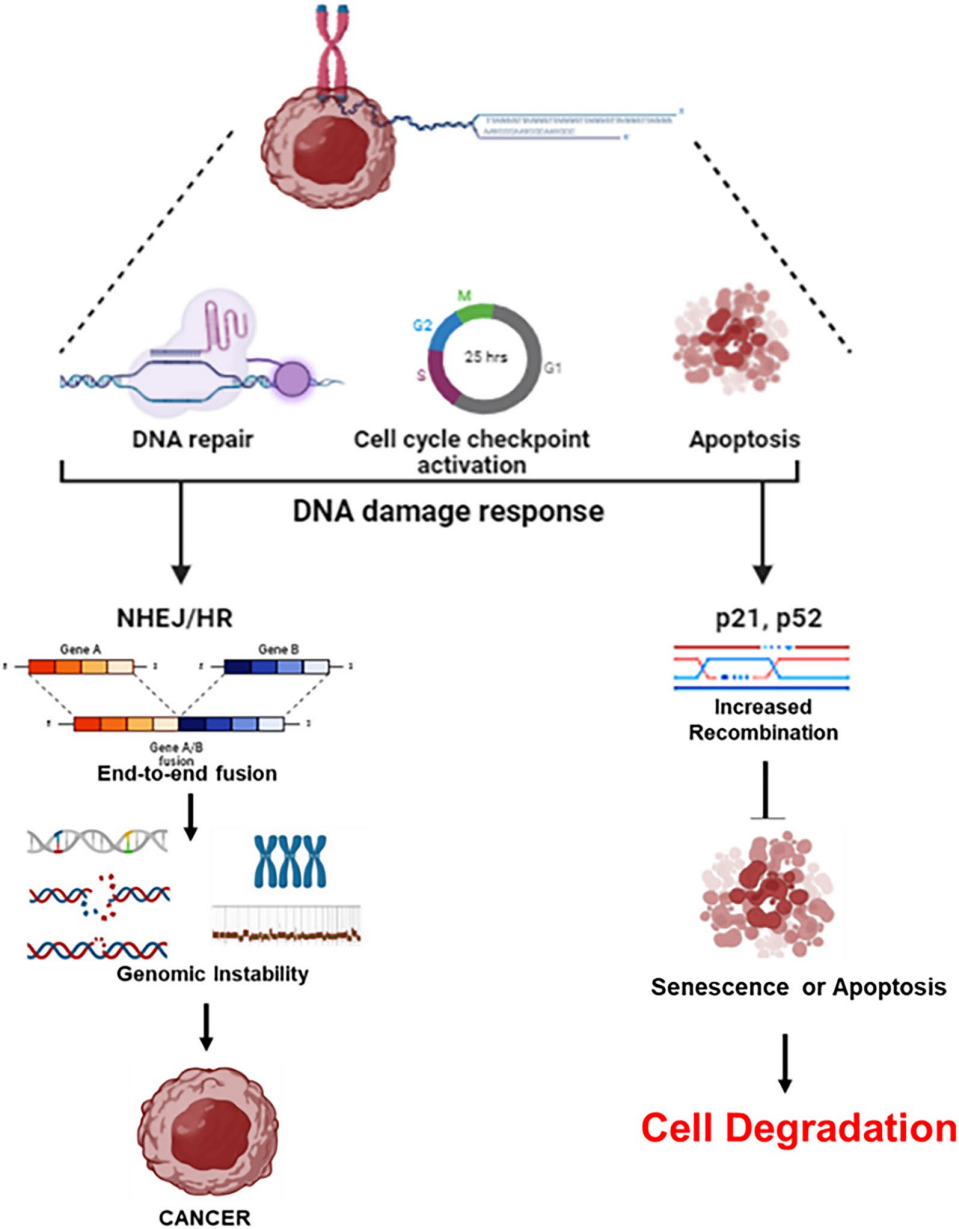
Telomerase dysfunction leads to Cancer: When a p53/p21-dependent cell cycle arrest occurs, the damage can be 'repaired' through either the non-homologous end-joining (NHEJ) pathway, resulting in chromosomal end-to-end fusions or the homologous recombination (HR) pathway, leading to telomere length changes and terminal deletions. Both pathways induce chromosomal instability, potentially leading to the amplification of oncogenes and the loss of tumour suppressor genes, thus increasing the risk of cellular transformation and the initiation of cancer Cellular Degradation: Activation of the tumour suppressor proteins p53 and/or p21 can lead to apoptosis or senescence of cells, resulting in tissue degeneration and, ultimately, organ failure

Excessive telomere shortening due to telomerase mutations or severe telomere uncapping caused by shelterin dysfunction can trigger a DNA damage response (DDR) at chromosome ends, mistaking them for double-strand breaks. This can activate the nonhomologous end-joining pathway, leading to chromosomal end-to-end fusions, while increased homologous recombination might cause rapid telomere length changes and terminal deletions [19]. Critically short telomeres fail to recruit enough shelterin to suppress checkpoint activation, a model supported by evidence that disrupting specific shelterin components like TERF1 can induce a DDR even without telomere shortening (Fig. 2). Telomere dysfunction, whether due to critically short telomeres or uncapping, triggers a DNA damage response (DDR) through the activation of upstream kinases such as DNA-PK, ATM (ataxia-telangiectasia mutated), and ATR (ataxia-telangiectasia and Rad3 related) [20].

Most CCs express telomerase, a protein that maintains telomere length, which is inactive in most normal cells. CCs preserve telomere length by preventing telomere shortening, allowing them to bypass replication limits and divide indefinitely, a key characteristic of cancer. Changes in the structure and function of shelterin complex proteins can promote tumorigenesis and cancer progression. Understanding shelterin complex biology suggests that targeting shelterin proteins could be a novel strategy for cancer therapy.

The messenger RNA (mRNA)-microRNA (miRNA)-long noncoding RNA (lncRNA) axis plays a crucial role in different cancer hallmarks, including inhibition of telomere shortening. This makes them potential biomarkers for predicting clinical outcomes, including survival in cancer patients. This study focuses on two types of noncoding RNAs (miRNAs and lncRNAs) that interact with differentially expressed metastatic mRNAs. The miRNAs, which are dysregulated in cancers, contribute to cancer hallmarks by modulating various pro- and anti-oncogenic properties. These small, conserved, endogenous RNA molecules [21] are 19–25 base pairs long and regulate gene silencing by binding post-transcriptionally to target mRNAs' 3'-untranslated (UTR) region. The lncRNAs are RNA transcripts typically longer than 200 nucleotides that are not translated into proteins [22].

Since time immemorial, plant-derived natural compounds and molecules have been exploited for their anti-oncogenic properties. Existing drugs, including chemotherapeutic agents, often have significant side effects, and prolonged use can lead to resistance in CCs. Minimizing side effects and preventing chemoresistance can be achieved through dosage adjustments or modifications of these drugs [23]. Even with dosage modifications, chemotherapeutic agents have demonstrated the ability to cause cytotoxicity and result in tumour regression. However, only a limited number of drugs specifically target tumour tissues. Increasing research indicates that the development and recurrence of cancer are influenced not only by physiological factors but also by psychological and social aspects of a patient's life.

Despite decades of extensive research aimed at developing novel therapies, effective treatment for cancer remains elusive due to the disease's complexity and heterogeneity. CCs often acquire drug resistance, and available treatments frequently come with severe side effects. Specifically, the study aims to discover novel alternative therapeutics and non-coding biomarkers, such as miRNAs and lncRNAs, that are significantly involved in maintaining telomeres for cellular immortality. This in-silico research may provide new targets for validation in experimental setups, potentially leading to more effective cancer treatments.

In a nutshell, in this study, we aim at the detection of significant cliques, biomarkers and drugs derived from interaction networks of mRNA–miRNA–lncRNA–phytocompound interactions, followed by a multi-omics analysis of the shelterin proteins that can be useful and critical for an enhanced understanding of the telomerase activity in different cancers, which may lead to the identification of newer therapeutic strategies as well.

### Methodology

#### Retrieval of significant mRNAs

Various research and review articles have extracted information about the shelterin protein complex with keywords such as ‘shelterin proteins’, ‘shelterin protein complex’ and ‘telomeric shelterin proteins in cancers’ [24]. Within this complex, TERF1 and TERF2 bind to double-stranded telomeric DNA, while POT1 binds directly to single-stranded DNA. TINF2 and TPP1 serve as bridges between TERF1, TERF2, and POT1, stabilizing the entire complex. Although TERF1, TERF2, and POT1 directly bind to telomeres, TINF2 and TPP1 indirectly interact with telomeres through their associations with TERF1 or TERF2 [25].

#### Retrieval of non‑coding RNAs

In this study, we focus on two types of non-coding RNAs: miRNAs and lncRNAs. We retrieved common miRNAs interacting with shelterin proteins from the ENCORI using the RNA-RNA menu for miRNA-RNA (https://starbase.sysu.edu.cn/), [26] miRTarBase (https://mirtarbase.cuhk.edu.cn/~miRTarBase/miRTarBase_2022/php/search.php) using the search option and then searching for miRNAs using the ‘By Target Gene’ option [27] and in TargetScan (http://www.targetscan.org/vert_80/) [28] database, the list of miRNAs can be downloaded by giving the input of the human gene symbol of the respective mRNA. The ENCORI database includes experimentally identified RNA–RNA and protein–RNA interaction networks derived from 108 CLIP-Seq datasets across 37 independent studies. miRTarBase focuses on miRNAs, and their high-throughput validated miRNA-target interactions, particularly supported by CLIP-Seq verified data. TargetScan predicts miRNA targets by examining conserved 8-, 7-, and 6-mer sites complementary to the miRNA seed region.

For common lncRNAs interacting with differentially expressed genes (DEGs), we utilized two databases: lncBase v3 for retrieving interacting lncRNAs for miRNAs by entering the miRNA of interest and selecting the specific tissue types) (For eg. hsa-let-7a-5p in bone tissue) (https://diana.e-ce.uth.gr/lncbasev3) [29]. ENCORI was also used to identify interactions between miRNAs and lncRNAs under the ‘miRNA-Target’ drop-down menu.

#### Retrieval of interacting small compounds

The drugs interacting with mRNAs are obtained from the Comparative Toxicogenomics Database (CTD) (https://ctdbase.org/) by entering the gene name in the tab ‘Search by gene’ under ‘Chemical–Gene Interaction Query’ [30]. CTD contains manually curated data on mRNA–drug interactions that affect various biological pathways implicated in diseases such as cancer. For information on drugs interacting with miRNAs, we utilized the Sm2miR database (http://www.jianglab.cn/SM2miR/) [31] using the miRNA name and species, which provides comprehensive data on drugs and small molecules that influence miRNA expression and miRNA-associated therapeutics. The lncRNA–drug interaction data is retrieved from the D-lnc database (http://www.jianglab.cn/D-lnc/) [32] by selecting ‘Homo sapiens’ under species and entering the lncRNA of interest in the ‘Search by lncRNA’ tab.

#### Generation of an mRNA–miRNA–lncRNA–drug interaction network, followed by hub RNA identification, module detection, and transcription factor analysis

To analyze the interactions within the complex network of miRNA–mRNAs–lncRNA–drugs, the Cytoscape v3.9.0 software [33] was employed. This freely accessible platform allows the visualization of intricate networks involving multiple biological entities. Using the 'Merge' attribute from the 'Tool' dropdown menu, smaller networks such as mRNA–mRNA, mRNA–lncRNA, mRNA–drugs, miRNA–drugs, lncRNA–drugs, and miRNA–lncRNA were consolidated. The Maximal Clique Centrality (MCC) ranking method from the 'cytohubba' plugin was then utilized to identify hub mRNAs within the interaction network. Additionally, the 'MClique' plugin was used to detect cliques [34].

The MCODE plugin [35] was applied to the interaction network to identify the top subnetworks with an MCODE score of 3 and above to discover significant transcription factors (TFs). The most prominent carcinogenic conditions were identified by analyzing the highly expressed hub genes (rank-wise: deep red denotes the most significance), with a cut-off degree of ‘30’ for significant genes from the topological parameters like ‘degree’, ‘betweenness’, ‘closeness’, ‘radiality’, ‘bottleneck’ and ‘eccentricity’. For mRNAs from the top two subnetworks identified by MCODE, interacting miRNAs were searched using the ENCORI database. Once the mRNAs and miRNAs were obtained, their interacting TFs were retrieved from databases like TRRUST (https://www.grnpedia.org/trrust/) corresponding to mRNAs under the ‘Search’ tab in the ‘Search a gene in TRRUST database’ [36] and TransmiR (http://www.grnpedia.org/trrust) for miRNAs by exploring the ‘Search’ tab, giving the miRNA name and organism name ‘Homo sapiens’ [37]. The subnetworks were merged to identify the hub TFs and the MClique plugin was employed on the interaction network, which involved mRNAs, miRNAs, and TFs. ChA3 TF analysis was conducted on all TFs obtained from TRRUST and TransmiR to validate the hub TFs identified through Clique analysis in MClique within Cytoscape. The significance of the TFs was further validated using ChA3 TF analysis [38].

#### Multi-faceted analysis of the shelterin proteins

##### Gene expression profiling

The TNMPlot database (https://tnmplot.com/) [39] offers expression profiles of shelterin proteins across 25 different types of cancer, utilizing nearly 57,000 samples from various RNA-seq and microarray datasets. It includes sensitivity/specificity plots based on expression cutoffs, illustrating the proportion of tumour samples with elevated expression compared to standard samples at different cutoff levels (minimum, first quartile, median, third quartile, maximum). These bar graphs provide clear insights into the clinical utility of a selected gene by highlighting its specificity to tumour cells, which is essential for identifying pharmacologically valuable targets. The expression profiles of these proteins are displayed cumulatively in box plots, and density graphs show the expression profiles of the shelterin proteins, comparing normal, tumour, and metastatic samples based on their log2 values.

###### Survival profiles related to the shelterin proteins

Additionally, we performed a survival analysis of mRNAs using Oncolnc (http://www.oncolnc.org/) [40], an interactive platform that links cancer patient survival data from TCGA with expression levels of mRNA, miRNA, and lncRNA, presented in Kaplan–Meier plots. Tumor samples from 503 patients were divided into high- and low-expression groups and analyzed using the log-rank test. The statistical significance of the selected markers was validated with a p-value < 0.05.

###### Shelterin protein complex-associated mutation profiles

Genetic mutations often impact more than just a single protein; they can influence the transcription of entire pathways. Comprehensive analysis of the mutational profiling of these proteins is carried out using Tumor Portal (http://www.tumorportal.org), cBioPortal (https://www.cbioportal.org/) [41] and muTarget (https://www.mutarget.com/) [42].

Tumor Portal facilitates the exploration of genes, cancers, mutations, and annotations by allowing users to delve into various tumour types, genes, and visual representations. cBioPortal provides mutational data based on the chromosomal positions of shelterin proteins. This integrative platform offers high-quality genetic profiles detailing various molecular alterations. This database identifies genetically altered mRNAs by examining samples from the TCGA dataset (Cell, 2015), revealing different chromosomal mutations, their positions, and base pair changes. In addition to the default layout, cBioPortal features supplementary bar plots adjacent to the heatmap, displaying the number of various modifications for each sample and gene. Mutational data from cBioPortal is then visualized using the web-based Circos tool (https://circos.ca/software/download/circos/) [43].

The muTarget database encompasses 18 solid tumour subtypes and 7800 patient samples, incorporating somatic mutations and RNA-seq gene expression data. Based on filters like ‘prevalence at least 3%’, ‘*p*-value cutoff < = 0.05’, ‘fold change cutoff = 1.44’ and ‘FDR = < 5%’, muTarget facilitates the identification of biomarkers and potential therapeutic targets across various types of solid tumours.

###### Gene correlation profiles

Correlation profiles for shelterin proteins are analyzed using the TIMER database (http://timer.cistrome.org/) [44], employing Pearson and Spearman correlation coefficients. These coefficients are widely used to measure relationships between variables: the Pearson correlation coefficient evaluates linear relationships, while the Spearman correlation coefficient assesses monotonic relationships [45, 46].

In the TIMER database, the Gene-Corr module investigates the correlation between a gene of interest and a list of other genes across various cancer types. The resulting heatmap displays the purity-adjusted partial Spearman's rho value, indicating the strength of their correlation. Users can select the 'Purity Adjustment' checkbox to adjust the association for purity.

###### O-linked Glycan prediction

Glycans play a critical role in cancer, serving as a potential source for developing new clinical biomarkers. Their ability to influence various stages of tumour progression and the biosynthetic pathways involved in glycan structures make them a promising target for cancer therapy. GlyGen is an open-source web interface that predicts shelterin-associated glycans by integrating glycoinformatics tools to explore glycoscience data. The GlyGen portal (https://www.glygen.org/) [47] currently includes N- and O-glycans from human, rat, and mouse species. In the database, from the drop down list of ‘Molecule’ the authors have chosen Glycan from the drop-down menu and under the ‘From ID type’ we need to select ‘PDB’ and then enter the PDB IDs of the shelterin proteins to retrieve the list of glycans corresponding to each one.

###### Functional enrichment analysis

An automated functional and pathway-enrichment analysis was performed on the shelterin protein cluster using hypergeometric testing by the STRING database (https://string-db.org/) [48]. The database performed overrepresentation tests for gene ontology (GO) terms such as biological process (BP), cellular components (CC), Molecular Functions (MFs), enriched tissues, and Reactome pathways. Statistically significant enriched terms with a *p*-value < 0.05 were considered along with the overlapping genes against a statistical background of the entire genome. The false discovery rate (FDR) is a measure that describes how significant the enrichment is. The p-values corrected for multiple testing within each category using the Benjamini–Hochberg procedure.

###### Gene cooccurrence profiling of shelterin complex

The STRING database retrieves the gene cooccurrence profile of the shelterin proteins across a wide range of taxonomy. In the STRING database, the lighter the red hue, the lesser the similarity or percentage of conservation of the proteins across the different taxonomies. Correlations of these presence/absence profiles can predict interactions. Clade coverage for groups of genomes collapsed in the phylogenetic tree, two distinct colours indicate the lowest and highest similarity observed within that clade.

**Fig. 3:**
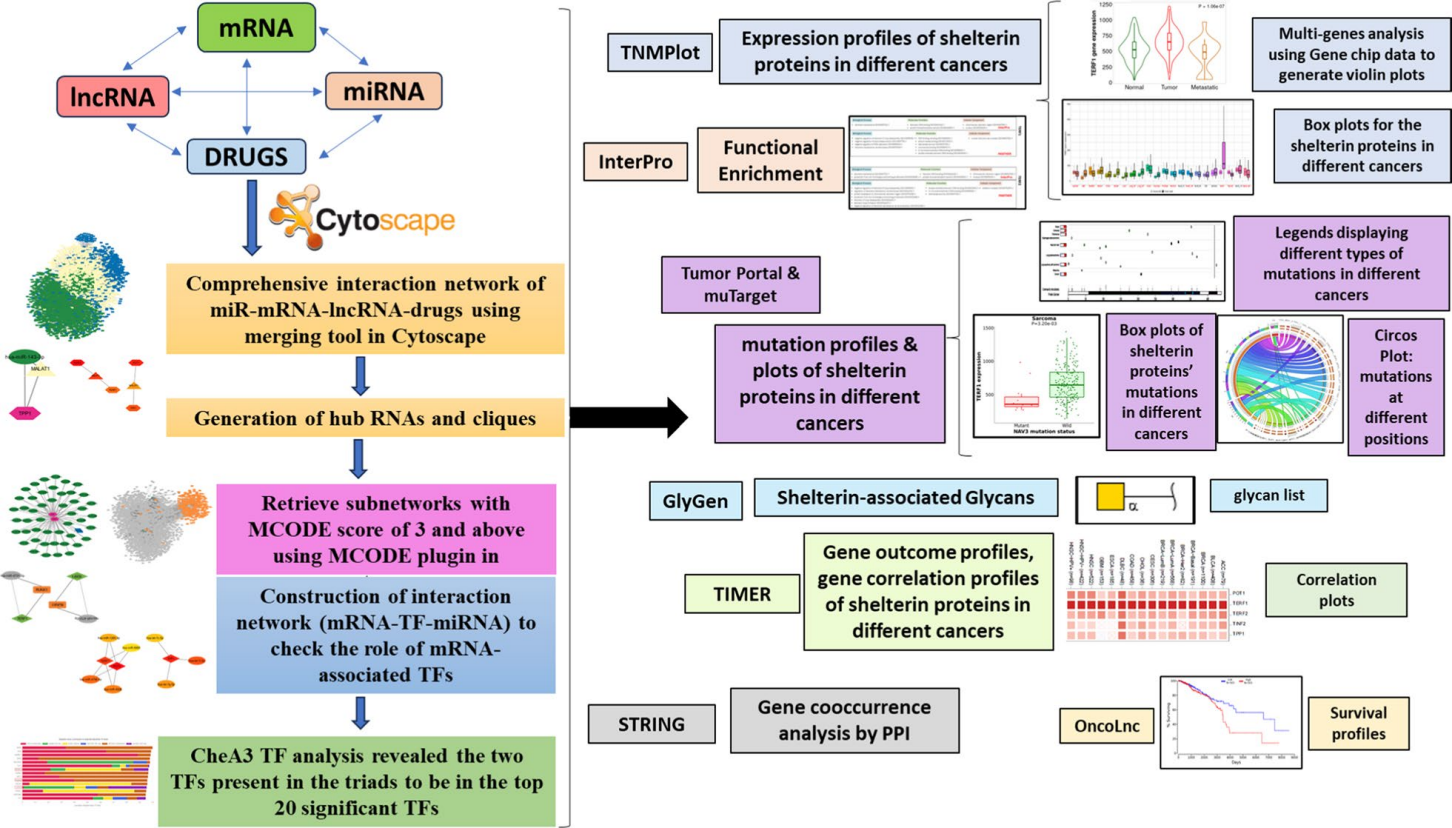
The fundamental analysis framework involved network pharmacology and multi-faceted analysis of the shelterin proteins

Figure 3 serves as a comprehensive guide, showcasing this study's systematic approach and methodology to achieve the research objectives. It emphasizes the sequential flow of tasks and the interconnections between different analysis components, ensuring a thorough understanding of the investigative process.

## Results

### Retrieval of interacting non-coding RNAs

For the 5 shelterin proteins, 2056 interacting miRNAs are retrieved from TargetScan and ENCORI databases and 1275 lncRNAs from (mRNA–lncRNA interaction data) and ENCORI (miRNA–lncRNA interaction data) are considered for the study. 549 small molecules interacting with mRNA, miRNA and lncRNA were discovered from CTDbase, Sm2miR and D-lnc database, respectively.

### Interaction network analysis using cytoscape v3.9

In Cytoscape, six distinct interaction tables are individually loaded to encompass different types of interactions: mRNA–miRNA, miRNA–lncRNA, mRNA–lncRNA, mRNA–small molecules, miRNA–small molecules, and lncRNA–small molecules. These tables are then merged to form a comprehensive interaction network, as depicted in Fig. S1. This network visually represents mRNAs, miRNAs, lncRNAs, and drugs by pink hexagons, green ellipses, yellow triangles, and blue parallelograms, respectively (Fig. S1).

**Fig. 4:**
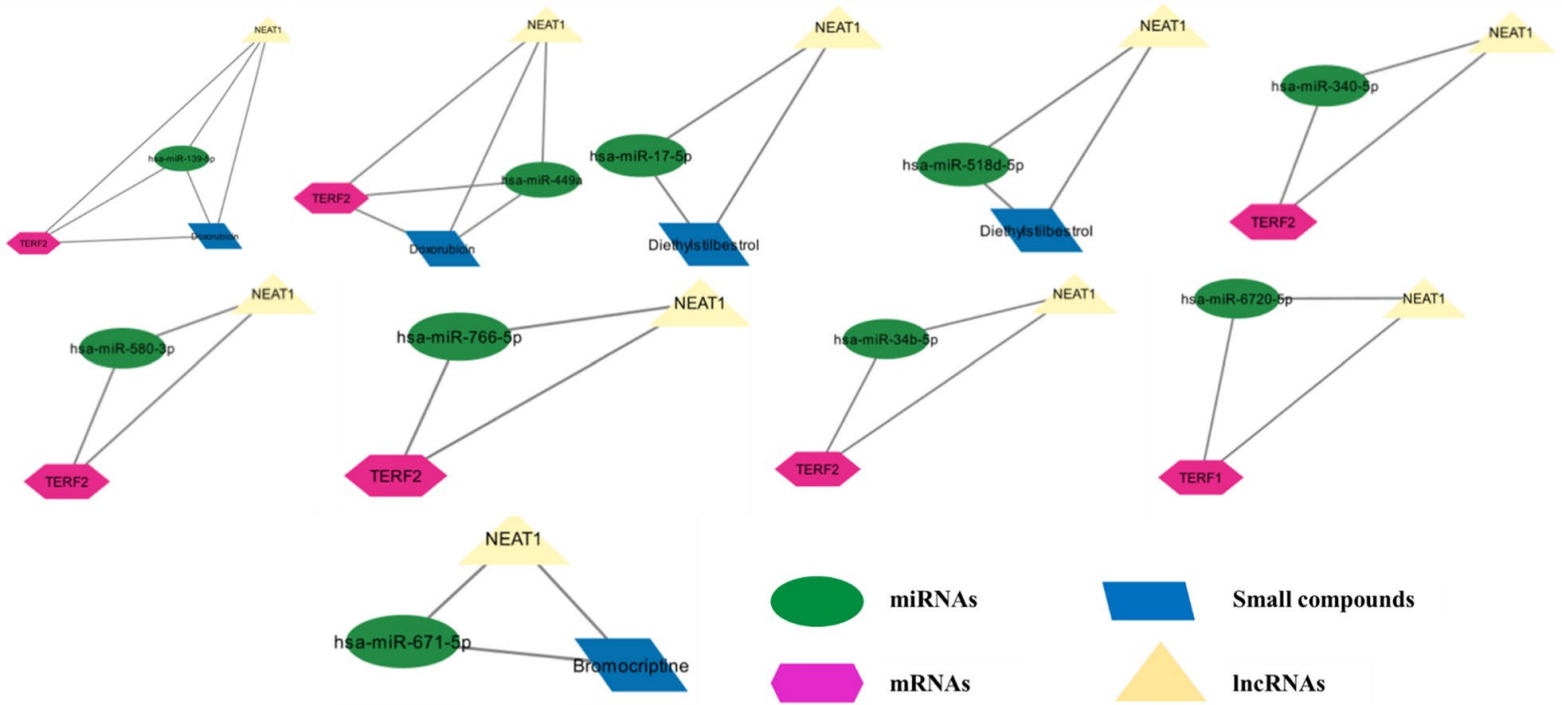
The top 10 cliques from the merged network. These cliques are retrieved using the MClique cytoscape plugin on the ‘Merged’ version for the interaction network of mRNA-miRNA-lncRNA-small compounds. The top 10 cliques depict the most closely interacting coding and non-coding RNAs with various small molecules

The CytoHubba and MClique plugins were utilized to analyze the interaction network. CytoHubba identified the top 10 hub RNAs, including all five shelterin proteins and NEAT1, MALAT1, XIST, SNHG14, and KCNQ1OT1. Additionally, the MClique plugin revealed a total of 567 cliques within the network: the two largest cliques contain four nodes each, while the remaining 565 cliques consist of three nodes each. Figure 4 illustrates the top 10 cliques. Examining the top 20 cliques, significant mRNAs identified include TERF1 and TERF2 and notable lncRNAs include NEAT1. No redundant miRNAs were found among these top cliques. Regarding drugs, doxorubicin and diethylstilbestrol appear in the two most prominent cliques. Furthermore, bromocriptine is present in one of the cliques.

### Retrieval of sub-networks from the interaction complex network

MCODE is implemented on the merged interaction network based on the haircut algorithm, considering parameters like a k core of 2, a node cut-off value of 0.2, and a maximum depth of 100. The clustering score was utilised to create sub-networks. Seven sub-networks were retrieved using the plugin with an MCODE score cut-off of 3. The sub-networks with MCODE scores of 3 and above are taken into consideration for this study. Table 1 lists the MCODE score of the seven sub-networks.

**Table 1:** The MCODE subnetworks with a score of 3 and above

Subnetwork ranking	MCODE score
1	6.154
2	3.918
3	3.36
4	3.429
5	3.429
6	3.25
7	3

### Construction of TF–miRNA–mRNA interaction network and TF analysis

**Fig. 5:**
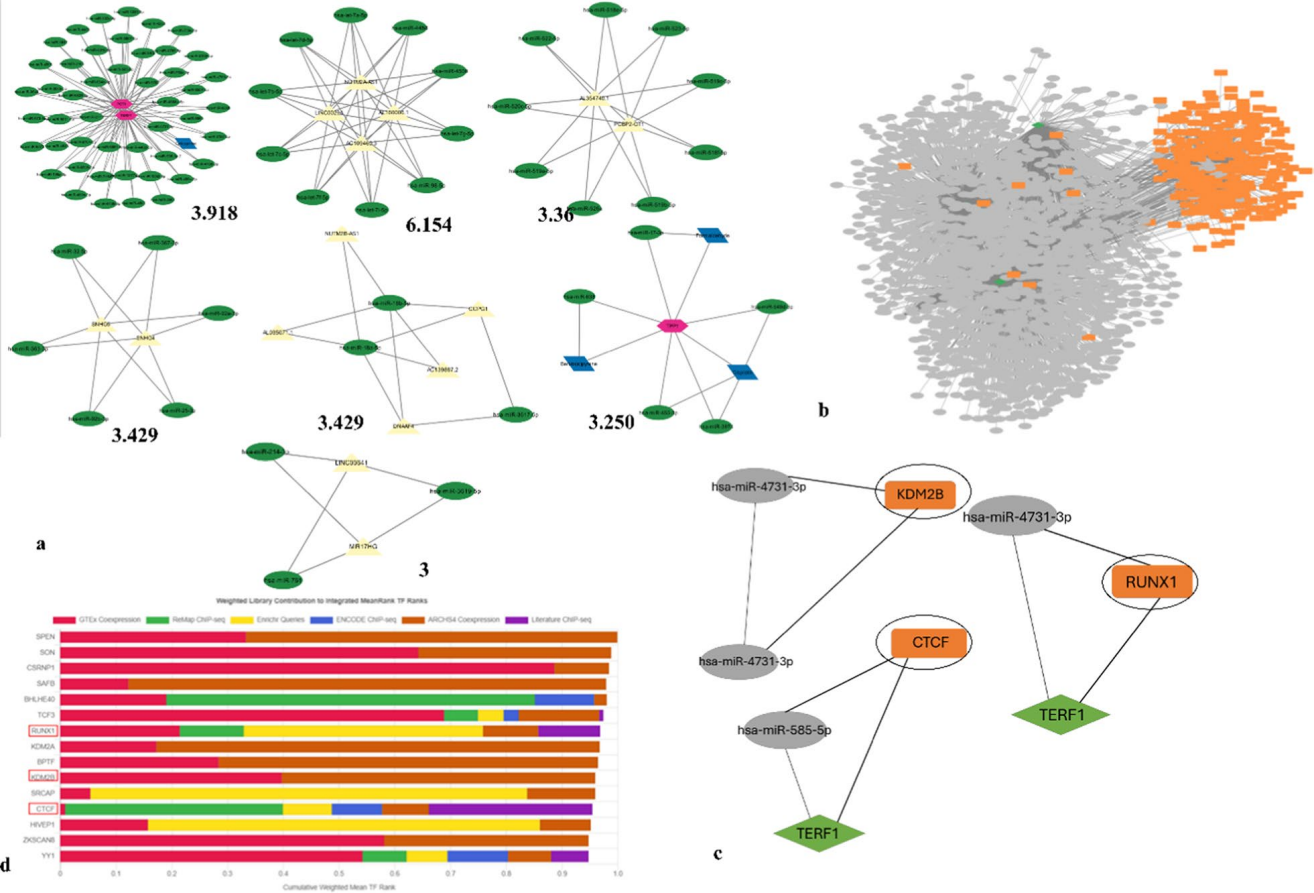
The MCODE results and TF analysis. **a.** The seven subnetworks with MCODE score cutoff of 3. **b.** The merged network comprises the three mRNAs-19 miRNAs-290 TFs **c.** The cliques generated from the network having the TFs. **d.** The CheA3 TF clustergram shows the significant TFs among the top 15

The mRNAs involved in these sub-networks (Fig. 5a) are further exploited to study a mRNA–miRNA–transcription factor (TF) interaction network. For the three mRNAs involved in the seven MCODE subnetworks, 19 interacting miRNAs, and 290 interacting TFs were retrieved. The merged network for mRNA–miRNA–TF is generated (Fig. 5b). Fig S2 represents the hub RNA network with the top 20 RNAs depicting the highly potential (based on colour ranking: red > orange > mustard > yellow) RNA biomarkers (POT1, TERF1 and TPP1). The MClique plugin of cytoscape is utilised to reveal that the TFs, RUNX1, CTCF and KDM2B are present in the top three generated cliques (Fig. 5c). Further, the CheA3 TF analysis reveals that these three TFs from the cliques are present among the top 15 integrated mean ranks TF analysis clustergram (Fig. 5d).

### Multi-factorial analysis of the shelterin proteins

#### Expression profiling of the shelterin proteins

**Fig. 6:**
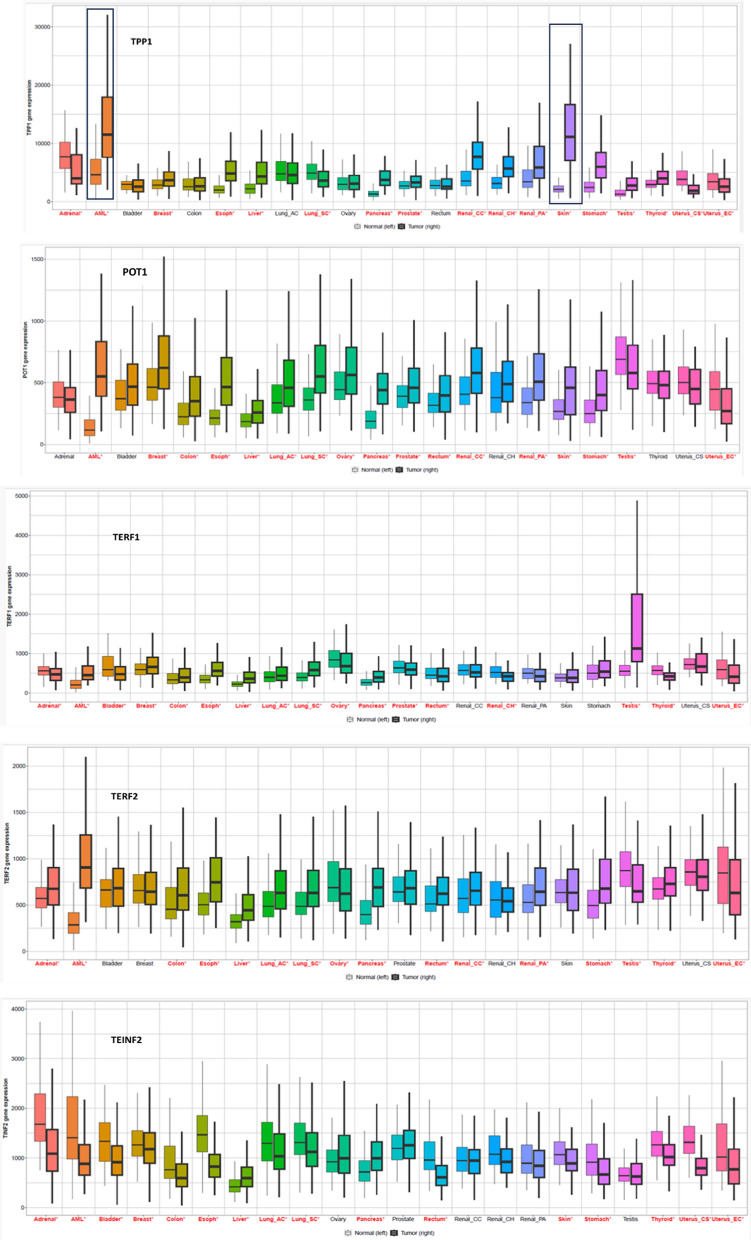
The box plots depict the expression analysis of the shelterin protein across 24 different cancers. TERF2 and TPP1 are most highly expressed in Acute Myeloid Leukemia (AML), followed by skin, prostate, breast, and renal malignancies, and oesophageal and ovarian cancers. TERF2 has its most expression in testicular cancer. POT1 seems to be expressed similarly in almost all cancers except hepatic cancer

The TNM plot represents the significance of enabling a real-time comparison of gene expression in ‘Normal’ Vs ‘Tumor’ of the shelterin proteins over a horizon of different cancers. TERF1, TERF2, TINF2, and POT1 are significantly expressed in testicular, Acute Myeloid Leukemia (AML), prostate and breast as well as renal cancers, respectively (Fig. 6). TPP1 is significantly expressed in AML and skin cancer. Additionally, Violin plots for individual cancers are portrayed as ‘Normal’ Vs ‘Tumor’ Vs ‘metastatic’ in Fig. S2 and listed in Table 2.

**Table 2:** Compared expression profiles of shelterin proteins across 12 different cancers with *p*-values

→ Cancer Protein	Breast ↓	Colon	Intestine	Kidney	Liver	Lung	Oesophageal	Oral	Ovarian	Pancreas	Prostate	Skin
TERF1	2.95e-14	3.17e-36	4.48e-01	7.7e-07	1.65e-36	1.16e-22	5.87e-03	2.39e-06	2.23e-07	1.06e-07	9.25e-05	4.19e-09
TERF2	1.71e-03	6.7e-08	4.08e-02	1.37e-07	7.15e-07	3.25e-18	7.04e-03	1.69e-02	1.27e-03	1.56e-07	4.15e-01	1.01e-09
TINF2	7.4e-27	9.32e-04	6.83e-01	5.39e-04	1.01e-07	3.14e-51	7.04e-12	9.54e-04	1.28e-06	7.38e-03	7.9e-04	4.8e-0.6
TPP1	1.31e-01	7.16e-10	1.27e-03	2.18e-01	6.4e-42	7.53e-09	1.09e-21	5.71e-07	1.62e-13	2.86e-02	1.91e-01	1.83e-31
POT1	8.5e-07	6.55e-71	2.12e-05	6.46e-35	2.89e-31	6.21e-41	3.26e-22	2.37e-08	4.67e-04	1.43e-09	4.6e-01	2.12e-18

#### Survival analysis of the shelterin proteins

**Fig. 7:**
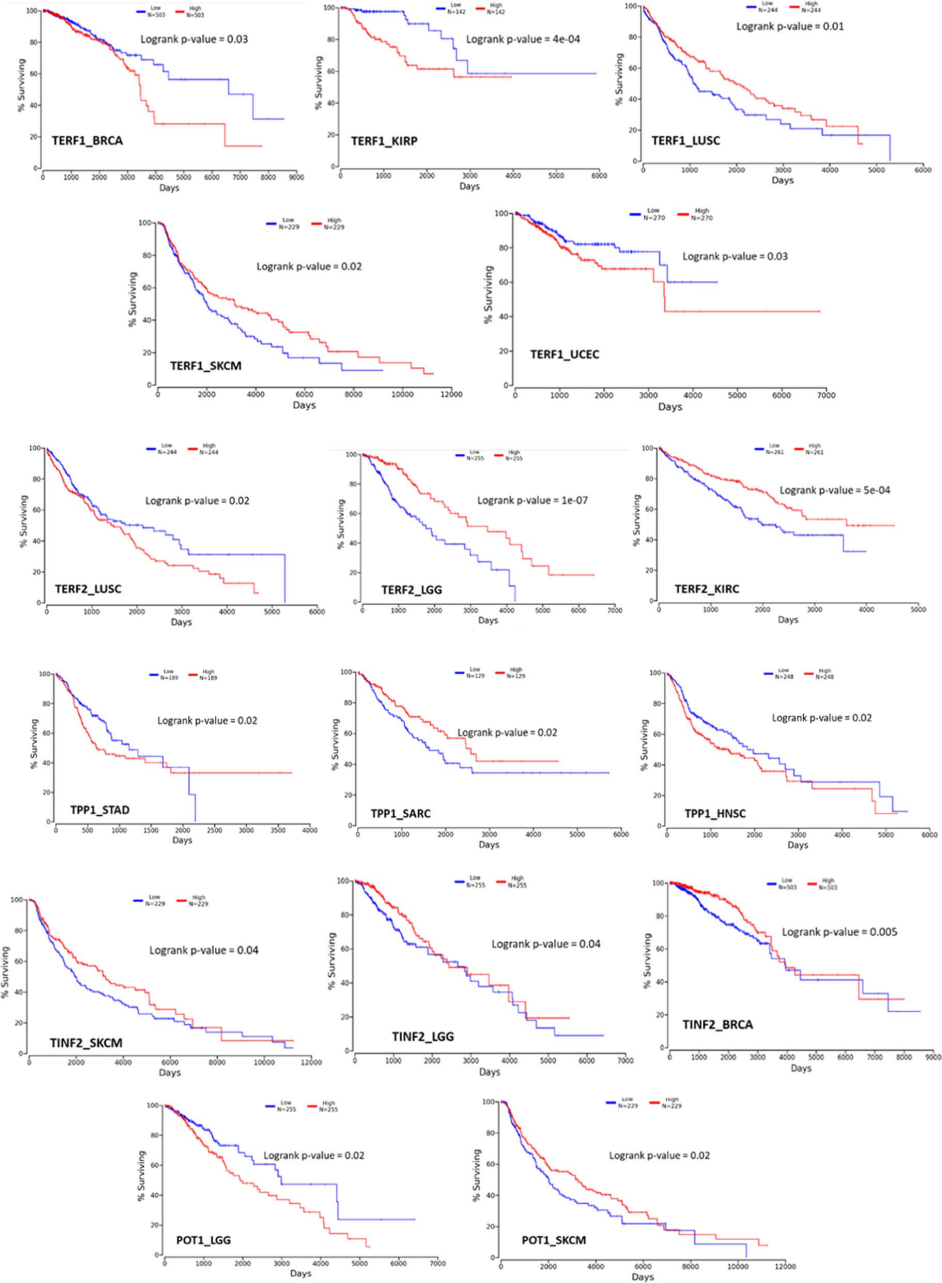
Kaplan-Mier plots of the patient survival of the **a.** TERF1 **b.** TERF2 **c.**TINF2 **d.** POT1

Unleashing an interactive platform, this innovation seamlessly weaves together survival data from TCGA with the intricate dance of mRNA, miRNA, and lncRNA expressions in cancer patients. Only markers with a resounding statistical significance, marked by a *P*-value < 0.05, made the cut. The shelterin proteins were assessed for survival analysis across various cancer datasets (Fig. 7).

The survival profiles of shelterin proteins in respective datasets are: TERF1 in BRCA (BReast CAncer gene), KIRP (Kidney renal papillary cell carcinoma), LUSC (Lung squamous cell carcinoma), SKCM (Skin cutaneous melanoma) and UCEC (Uterine Corpus Endometrial Carcinoma) datasets; TERF2 in LUSC, LGG (Low-grade glioma) and KIRC (Kidney renal cell carcinoma) datasets; TINF2 in LGG, BRCA and SKCM datasets; TPP1 in STAD (Stomach adenocarcinoma), SARC (Sarcoma) and HNSC (Head-Neck Squamous Cell Carcinoma) datasets and POT1 in LGG and SKCM datasets are found to be significantly associated with patient overall survival potential. Hence targeting these respective proteins in respective cancers has the potential of improving overall patient survival.

#### Mutation or polymorphic profiling of the shelterin proteins

**Fig. 8:**
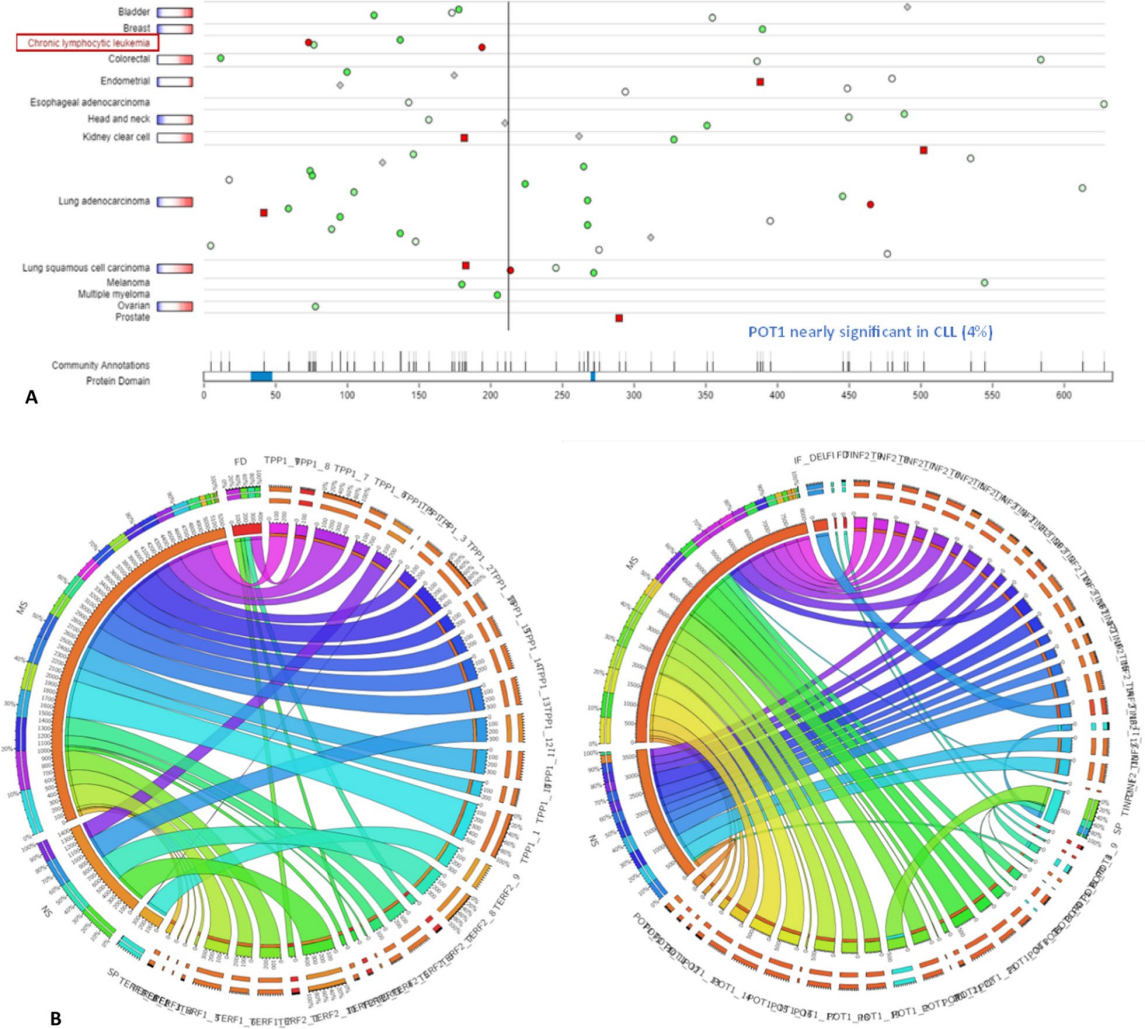
The mutation plots using **a**. POT1 is found to be significantly mutated in CLL. **b.** The circos plot represents the mutations associated with shelterin proteins at different chromosomal positions

The mutational profiling of the shelterin proteins is assessed via three different datasets from three publicly available databases. The Tumor Portal database provides plots that annotate mutations associated with individual proteins as different legends. POT1 has a nearly significant mutation profile in Chronic lymphocytic leukaemia (Fig. 8a).

The circos plot for all the mutations associated with the shelterin proteins at different chromosomal positions (Fig. 8b) is listed in Table S1. The muTarget database represents mutations associated with cancers like bladder cancer, breast cancer (TCGA), cervix cancer, colon adenocarcinoma, head and neck cancer, renal clear cell carcinoma, renal papillary carcinoma, brain lower grade glioma, liver cancer, lung adenocarcinoma, lung squamous carcinoma, ovarian cancer, prostate cancer, sarcoma, multiple myeloma, melanoma, gastric cancer, Thyroid cancer and Uterine cancer that includes all somatic mutations (Fig. S3).

#### Gene-correlation

**Fig. 9:**
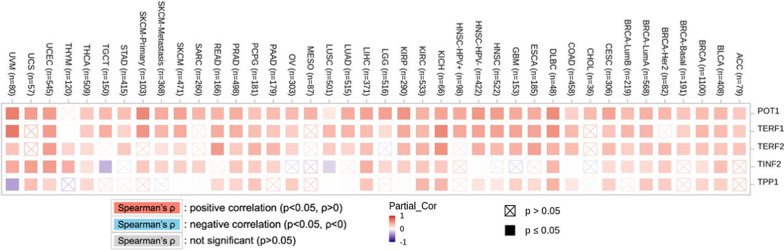
The gene correlation heatmap represents the degree of correlation

In the TIMER database, the correlation among the shelterin proteins is determined based on the cut-off of p-value of 0.05. TERF1 is found to be significantly correlated with the highest number of cancers. All five proteins are found to be nearly significantly correlated as per DLBC (n = 48), PAAD (n = 179) and PCPG (n = 181) datasets. TERF1, TERF2 and POT1 have a ‘near significant’ correlation in THCA (n = 509) and THYM (n = 120) datasets. TERF1 and POT1 are significantly correlated as per UVM (n = 80) datasets. TINF2 is negatively correlated based on the TGCT (n = 150) and LUSC (n = 501) datasets, while TPP1 is also negatively correlated as per the UVM dataset (Fig. 9).

#### Glycans

**Fig. 10:**
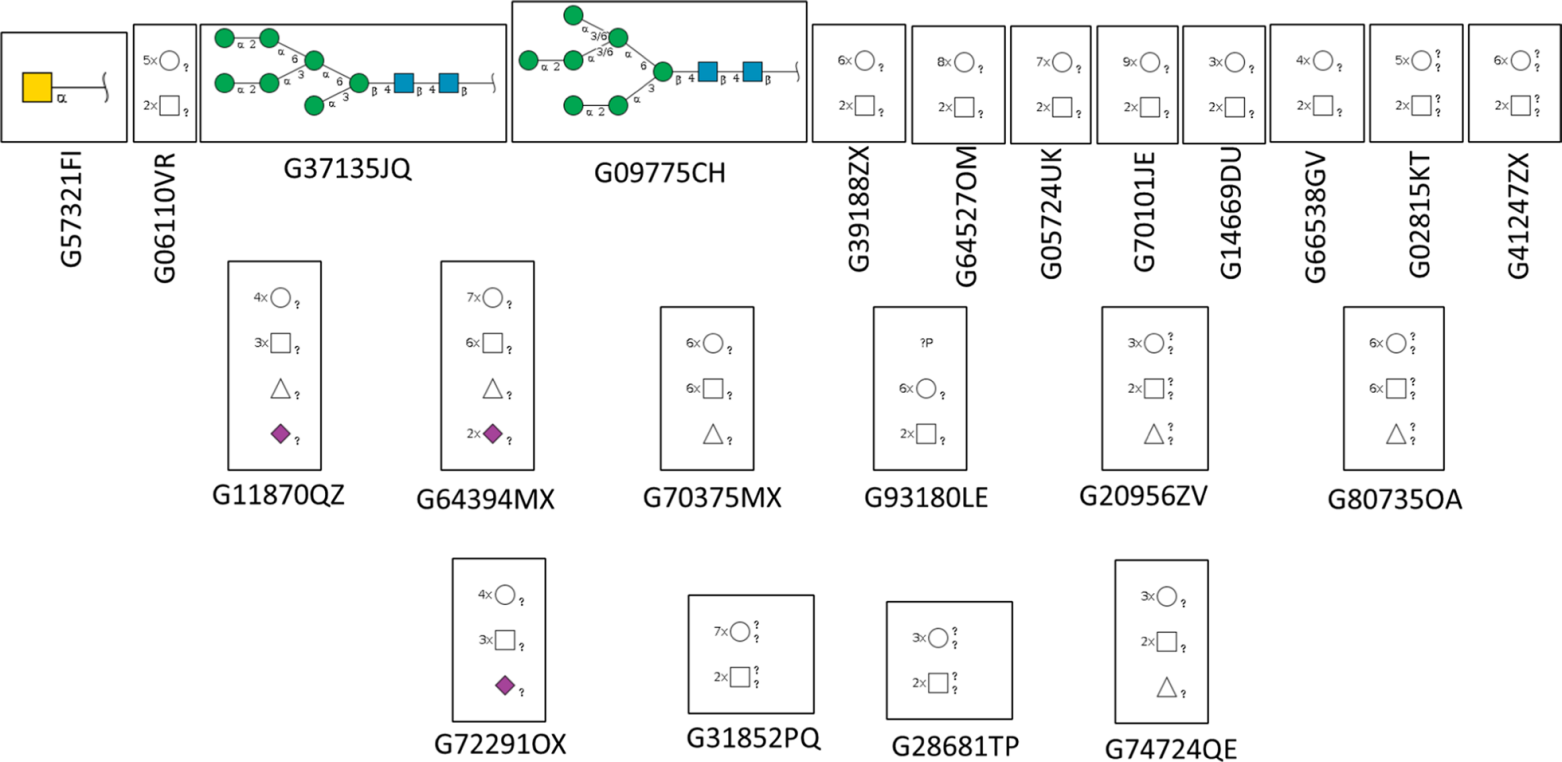
The glycosylated domains associated with the only shelterin protein, TPP1. The squares represent Nacetyl Hexosamine (GlcNAc), the circle galactose, the rhombus/diamond sialic acid, and the triangle fucose. The colour coding of each shape signifies a particular type of monosaccharide or alteration. Yellow and blue squares frequently represent N-acetylgalactosamine (GalNAc), whereas green circles typically represent mannose. α and β represent the anomeric conformation of the glycosidic bond

The functional significance of O-linked glycosylation for the immunosuppression role of TPP1 has encouraged us to check and analyze the probable glycosylation sites in TPP1. This is important because the variations in glycosylated TPP1 can be a significant factor while designing cancer therapeutics against TPP1 for different cancers. Among the five shelterin proteins of interest, TPP1 is the only one with 22 glycosylated domains (Fig. 10).

The squares represent N-acetyl Hexosamine (GlcNAc), the circle galactose, the rhombus/diamond sialic acid, and the triangle fucose. The colour coding of each shape signifies a particular type of monosaccharide or alteration. Yellow and blue squares frequently represent N-acetylgalactosamine (GalNAc), whereas green circles typically represent mannose. α and β represent the anomeric conformation of the glycosidic bond. The symbols α and β indicate alpha and beta linkages, respectively. Numbers 2, 3, 4, and 6 represent the location of the glycosidic bond on the sugar ring, indicating how one sugar binds to another. For example, "α2" or "β4" indicate the link between specific carbon atoms of two sugars. Multipliers (×) represent the amount of a given monosaccharide in a glycan structure. '?' most likely denotes an unknown or unidentified component of the structure. For example, if it occurs adjacent to a form, it might indicate that the kind of monosaccharide or linkage is unclear. The letter 'p' is not conventional in glycan notation and may be context-specific. It might represent a phosphate group or another change if compatible with the diagram's context. Some glycan structures are branching. Glycans frequently have branched structures due to numerous connections, and branching is necessary for their function in biological systems. Branching patterns can potentially alter glycan function, notably in cell signalling and chemical recognition. In the case of TPP1, the first three are the only fully defined glycosylation sites. The first motif is Tn antigen with enzymes such as GALNT2,1,3,6,17,18,5,7,10,13,12,15,16,4,6,11,14 and 9. The second one has a composition of Hex7 HexNAc2 and enzymes like ALG3,14,1,12,2,9,13 and DPAGT1. The last one has ALG11,14,1,2,13 and DPAGT1 as enzymes.

G74724QE, G92275SC, G14669DU, G28681TP, G34989PA, G01485JJ and G20956ZV are O-glycans, while glycosphingolipids are G02815KT, G06110VR, G00912UN, G74724QE, G92275SC, G14669DU, G66538GV, G72291OX, G28681TP, G50282JC, G84349RE and G20956ZV. All but G57321FI and G93180LE are N-glycans.

#### Functional enrichment

**Fig. 11:**
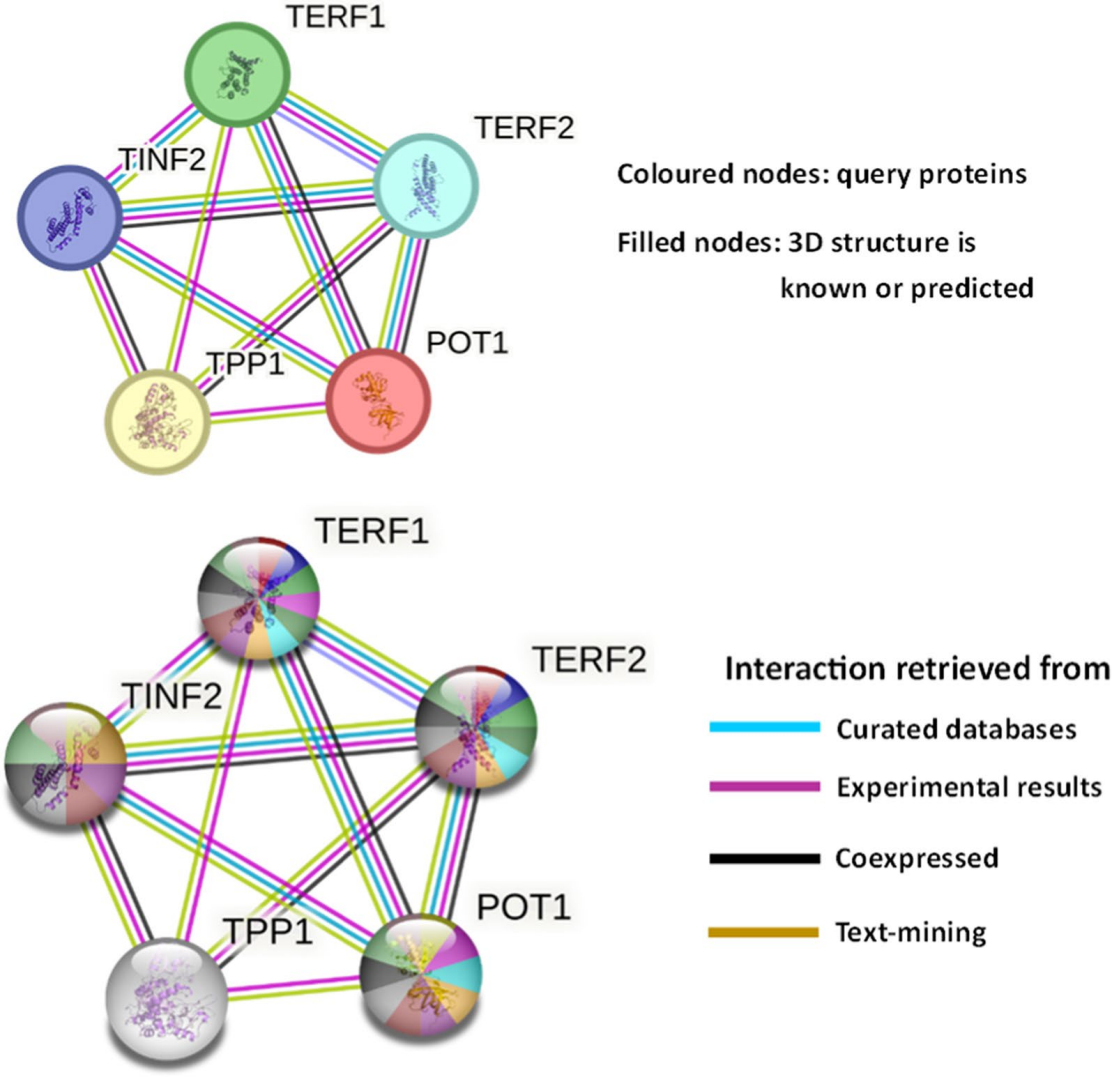
Functional enrichment analysis associated with the shelterin protein complex

The InterPro database lists the functional gene set enrichment based on InterPro and PANTHER analysis. Figure 11 represents the PPI and Table S2 lists the detailed information regarding the same.

#### Conservation profile of shelterin proteins

**Fig. 12:**
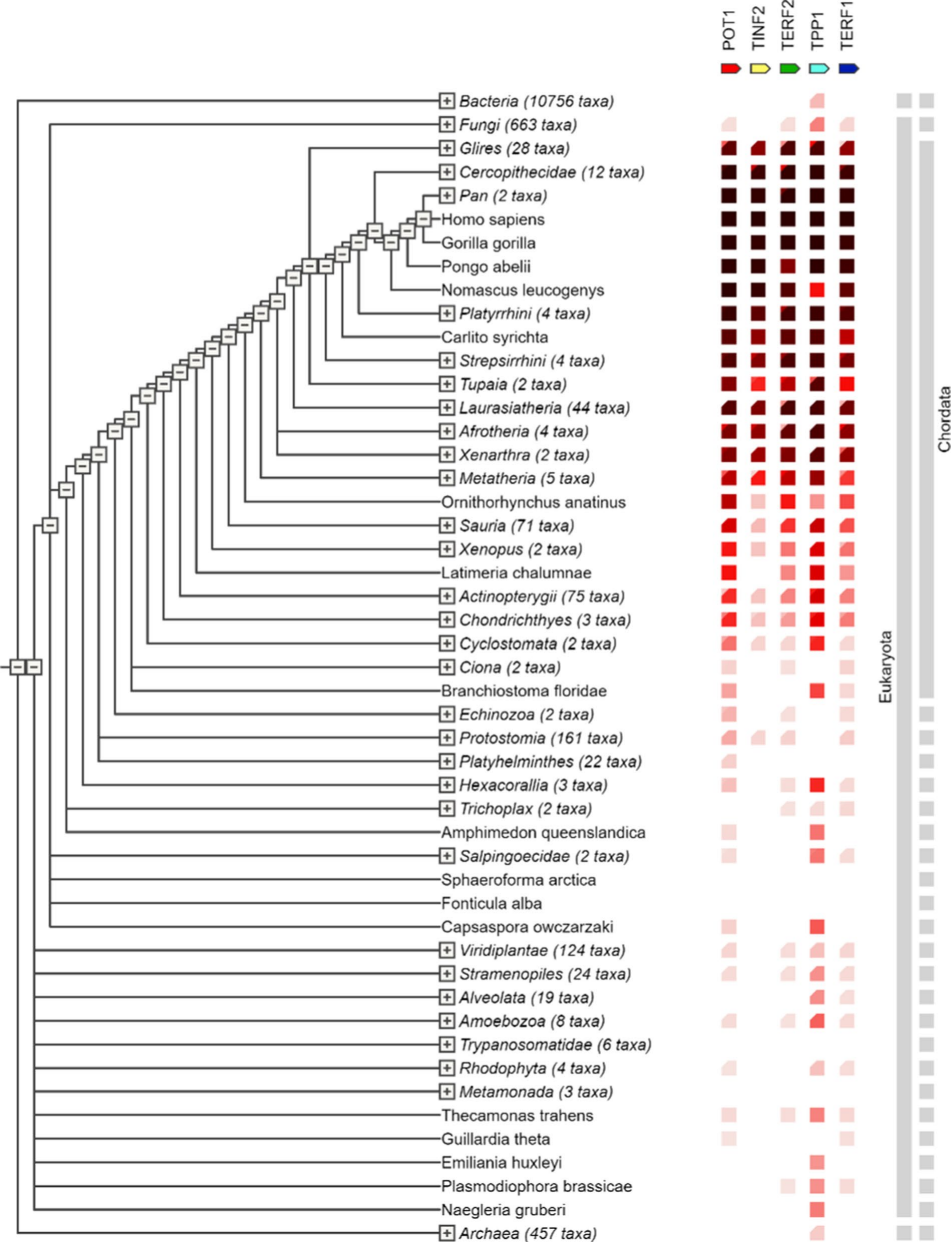
The co-occurrence heat-map visualisation of the shelterin proteins across different taxa

Utilising the STRING database, the co-occurrence profiling of the shelterin proteins was performed across a wide array of taxa. It is seen that the sequences of the proteins are most conserved among the taxa: Glires (28 taxa), Cercopithecidae (12 taxa), Homininae (4 taxa) [Pan (2 taxa), Homo sapiens, Gorilla gorilla], Pongo abelii, Nomascus leucogenys, Platyrrhini (4 taxa). Only TPP1 was less conserved in Nomascus leucogenys (Fig. 12). The darker shade of red the cooccurrence profile shows, the more significant it is.

## Discussion

Shelterin is a well-known protein complex linked to cancer, crucial for understanding tumorigenesis and developing telomere-based therapies. Recent research suggests that shelterin components significantly impact cancer and ageing, with mouse models supporting this theory. However, the full complexity of telomere capping structures remains elusive. Little is currently understood about the regulation of shelterin components during development and in pathological conditions. Changes in several shelterin components, such as TERF2, TERF1, and TINF2, have been identified in human cancers, highlighting potential therapeutic targets to combat cellular ageing and telomerase activity in various cancers.

Traditionally, therapies target individual signalling pathways or dysregulated proteins. This study focuses on the shelterin complex's role in mediating the function of various non-coding RNAs (ncRNAs) involved in cancer development. We employed network-based approaches to identify specific cliques involving the interactions of differentially expressed mRNAs, miRNAs, lncRNAs, and drugs/small molecules. These interactions provide a comprehensive view of the disease environment under the influence of interacting RNAs and drugs, suggesting new therapeutic strategies. The ncRNAs are often overlooked or treated as separate entities in disease mechanisms. However, they interact with mRNAs and play a crucial role in disease progression. For example, in the absence of a drug, dysregulated levels of ncRNAs can alter coding RNAs or protein expression levels, leading to disease. Using this network-based approach, we aim to understand the crosstalk between mRNAs and ncRNAs, paving the way for future therapeutic interventions considering the entire disease environment.

As discussed previously, conventional drugs are linked to various side effects and the development of drug resistance in cancer cells (CCs). These complications arise partly due to interactions with approved medications. In this context, not only messenger RNAs (mRNAs), microRNAs (miRNAs), and long non-coding RNAs (lncRNAs) have emerged as promising therapeutic targets, but also three key transcription factors (TFs) identified among the top 15 in ChEA3 analysis have shown significant potential. Targeting these specific TFs could be crucial in combating cellular ageing across different types of cancers. Focusing on these non-coding RNAs (ncRNAs) and transcription factors makes fine-tuning conventional cancer drug dosages possible. This approach aims to minimize adverse effects and enhance the overall effectiveness of cancer therapies. The miR139-5p is a prognostic thyroid cancer marker involved in HNRNPF-mediated alternative splicing [49]. The miR449a has evidence of being a prognostic biomarker in different cancers [50]. The miR17-5p is associated with poor prognosis in prostate cancer [51]. miR518d-5p utilizes mitochondrial activity to overcome liver tumour cell apoptosis [52]. miR340-5p targets RhoA, inhibiting colon cancer cell viability and metastasis [53]. The miR5580-3p inhibits oral cancer cell immortality by suppressing LAMC2 [54]. miR671-5p is involved in cellular immortality by targeting ALDH2 [55].

This study's network pharmacology approach reveals significant interactions between nine microRNAs (miRNAs) and the long non-coding RNA (lncRNA) NEAT1 with shelterin proteins. NEAT1 engages with TFs or shelterin complexes to activate the expression of oncogenes or cell cycle-regulating genes. NEAT1 promotes the process of EMT, which is linked to metastasis and cancer cell invasiveness. NEAT1 may affect the shelterin complex's ability to regulate telomerase activity. In certain malignancies, telomerase is activated to forestall telomere shortening, allowing limitless cell division. Interactions between NEAT1 and shelterin may impact this balance, resulting in telomere extension and the avoidance of cellular senescence, which is a crucial aspect of cancer cell longevity. If NEAT1 affects normal shelterin activity, it may cause impaired telomere function and chromosomal imbalances, which are common in cancer cells. Chromosomal instability can cause genetic diversity in tumors, which contributes to cancer growth and resistance to treatments. NEAT1 may regulate TERC (telomerase RNA component) and other telomere-associated parameters. The functions of NEAT1 in paraspeckle formation and shelterin in DNA damage response may overlap. NEAT1 regulates paraspeckles, which store stress-related substances. NEAT1 inhibitors could potentially increase the efficiency of telomerase inhibitors.

Shelterin proteins are crucial for the protection and maintenance of telomeres, the ends of chromosomes, and their interaction with these non-coding RNAs (ncRNAs) suggests that the ncRNAs may modulate the function of shelterin proteins. The modulation of these ncRNAs in shelterin proteins is further substantiated by evidence showing that conventional cancer drugs such as doxorubicin and diethylstilbestrol influence these ncRNAs. This indicates a complex interplay where these drugs not only target cancer cells directly but also alter the expression and function of ncRNAs, which in turn can modulate the shelterin proteins.

Significant transcription factors (TFs) like RUNX1, CTCF, and KDM2B have also been identified as potential biomarkers due to their interactions with miRNAs and shelterin proteins. These TFs play a crucial role in gene regulation and are involved in various cellular processes, including cell growth, differentiation, and apoptosis. The interactions of these TFs with miRNAs and shelterin proteins further highlight their importance in regulating telomere maintenance and cellular ageing. RUNX1, for example, regulates gene expression in hematopoiesis and has been implicated in several cancers. CTCF is a key regulator of chromatin structure and gene expression, playing a role in maintaining genome stability. KDM2B, a histone demethylase, is involved in chromatin remodelling and gene expression regulation [56–58].

Point mutations in POT1, particularly those affecting its oligonucleotide/oligosaccharide-binding (OB) fold domain (POT1 c.1432G > T (p.Cys476Phe) and c.1166C > T (p.Ala389Val), have been found in malignancies including chronic lymphocytic leukaemia (CLL), melanoma, and gliomas. Mutations in POT1 impair its capacity to bind telomeres, resulting in deprotected telomeres and high telomerase activity. POT1 interacts with telomeric Repeat-containing RNA (TERRA) to control telomere length. When POT1 is mutated, TERRA levels rise, resulting in telomere disruption, linked to various cancer types [13, 59]. Deletions and point mutations (R425W) in TRF1 have been found in malignancies, including lung cancer, glioma, and acute myeloid leukaemia (AML). TRF1-deficient cells have weak telomeres prone to breaking and recombination, promoting mutation and cancer growth. RF1 mutations may attract NEAT1 and MALAT1 to create paraspeckles that control alternative splicing and RNA processing in response to telomere disruption. Mutations such as TRF2 T241A reduce its ability to prevent telomeres from being detected as DNA breaks. Point mutations (K280E) in TIN2 have been found in patients with dyskeratosis congenita (DC), which is linked to an elevated cancer risk. TPP1 mutations (D224A) are linked to family melanoma, aplastic anaemia, and glioblastoma [24, 60–67].

By understanding these interactions, researchers can better grasp how ncRNAs and TFs contribute to telomere maintenance and the overall regulation of cellular ageing and cancer progression. This knowledge could lead to developing novel therapeutic strategies targeting these ncRNAs and TFs, potentially enhancing the efficacy of existing cancer treatments and reducing side effects by enabling more precise modulation of drug dosages.

The expression profiles reveal that shelterin proteins are prominently present in several major types of cancer, including acute myeloid leukaemia (AML), prostate, breast, renal, testicular, and skin cancers. Analysis using violin plots, which depict the distribution of data points, indicates that shelterin proteins are significantly expressed not only in normal and tumour states but also in the metastatic states of these cancers.

Furthermore, overall survival profiles indicate a strong association between the expression of all five shelterin proteins and patient survival across multiple cancer types, such as renal, breast, head and neck, lung, uterine, and stomach cancers. This correlation suggests that higher or altered levels of shelterin proteins can impact patient prognosis.

Mutations or variations in shelterin proteins are known to disrupt various cancer-related pathways. These disruptions can lead to dysregulated cellular processes, including accelerated telomere lengthening, which supports the concept of cellular immortality—a hallmark of cancer progression. Therefore, specific mutations in shelterin proteins are significant contributors to cancer tumorigenesis.

The conservation of shelterin proteins—a highly conserved complex across eukaryotic species—plays a crucial role in maintaining telomere integrity and chromosomal stability in normal cells. This evolutionary conservation ensures that the fundamental mechanisms of telomere protection and replication are maintained, preserving genomic stability across diverse cell types and organisms. The heatmap generated in the analysis highlights these overlaps, where darker shades represent genes involved in multiple cancers. Except for TINF2 in lung and testicular cancers and TPP1 in uterine cancers, shelterin proteins correlate positively across all 40 cancer types analyzed. The conservation of shelterin proteins is critical for telomere integrity and genomic stability in normal cells. These proteins, which include TRF1, TRF2, POT1, TIN2, TPP1, and RAP1, prevent telomeres from being identified as DNA damage, regulate telomerase activity, and prevent ineffective DNA repair mechanisms. This ensures controlled cell division in normal cells while also preventing premature senescence or apoptosis. However, in malignant cells, mutations or changed expression of shelterin components compromise telomere protection, resulting in genomic instability, a cancer hallmark. Mutations in proteins such as POT1 or TRF2 can cause excessive telomerase activity or telomere shortening, resulting in uncontrolled cell proliferation and chromosomal rearrangements that promote cancer. Thus, whereas shelterin complex conserved functions are critical for normal cell biology, their dysregulation in cancer drives oncogenesis.

The study by Luo et al. (PMID: 33,497,432) [68] provides a comprehensive pan-cancer analysis of shelterin proteins, investigating their expression patterns, mutations, and prognostic value across multiple cancer types. POT1, TRF1, and TRF2 were generally upregulated in most cancers. Frequent mutations in shelterin genes result in several cancers, including melanoma, lung adenocarcinoma, and colorectal cancer, especially POT1 mutations are prevalent. High expression of TRF2 and TIN2 was correlated with worse survival outcomes in lung cancer and glioblastoma, suggesting that shelterin protein levels can have prognostic significance. In this study, the comprehensive examination of shelterin proteins underscores their critical role in cancer biology. Their significant expression in various cancer states and their association with patient survival point to their potential as biomarkers for prognosis and therapeutic targets. The genetic correlations observed across different cancers suggest a common underlying mechanism involving shelterin proteins, further emphasizing their importance in cancer research and treatment strategies.

Glycosylation, the most intricate post-translational modification of proteins, plays a crucial role in cancer pathogenesis and progression. Altered glycans on the surfaces of both tumour and host cells and within the tumour microenvironment have been identified as crucial mediators of critical events in these processes. According to Ogata et al., malignant cells are notably more enriched with highly branched complex-type N-linked sugar chains compared to their regular counterparts [69]. TPP1's unique status as the only shelterin protein with 22 different glycosylation sites across various cancers highlights its potential significance in cancer biology. The modification of TPP1 through glycosylation likely plays a critical role in telomere maintenance, protein stability, cellular localization, and the aggressive characteristics of cancer cells, making it a promising focus for further research and therapeutic development.

## Conclusion

Current oncogenesis research is increasingly focusing on the shelterin complex. Various studies have identified the involvement of shelterin proteins in different tumours, including lymphocytic leukaemia, squamous cell carcinoma, non-small cell lung cancer, and breast cancer. Notably, the expression levels of certain shelterin components, such as TRF2, TRF1, and TIN2, have been found to be altered in many cancers. Some shelterin proteins also play crucial roles as either telomerase recruitment factors (POT1 and TPP1) or negative regulators of telomere length (TRF1 and TRF2). Understanding the role of the shelterin complex in telomere maintenance during tumorigenesis is essential. The data discussed underscore the potential of shelterin components as attractive targets for future anti-cancer therapies, offering promising prospects for prognosis and the prediction of cancer aggressiveness. Given their connection with telomere and telomerase biology, shelterin proteins could be pivotal in developing combined strategies and synergistic therapeutic interactions for cancer treatment.

## Supplementary Information

Additional file 1

## Data Availability

The authors confirm that the data supporting the findings of this study are available within the article, and its supplementary and publicly available data sources have been mentioned in the manuscript. For ex. https://tnmplot.com/, http://www.oncolnc.org/, http://www.tumorportal.org, http://timer.cistrome.org/, https://www.cbioportal.org/, https://circos.ca/software/download/circos/
