# Supplementary material for: Combatting cellular immortality in cancers by targeting the shelterin protein complex
Source: Biol Direct. 2024 Nov 22;19:120. doi: 10.1186/s13062-024-00552-4 (PMC11585132; doi:10.1186/s13062-024-00552-4)
Supplement: Supplementary file 1 — Additional file 1 [file 13062_2024_552_MOESM1_ESM.docx]

**
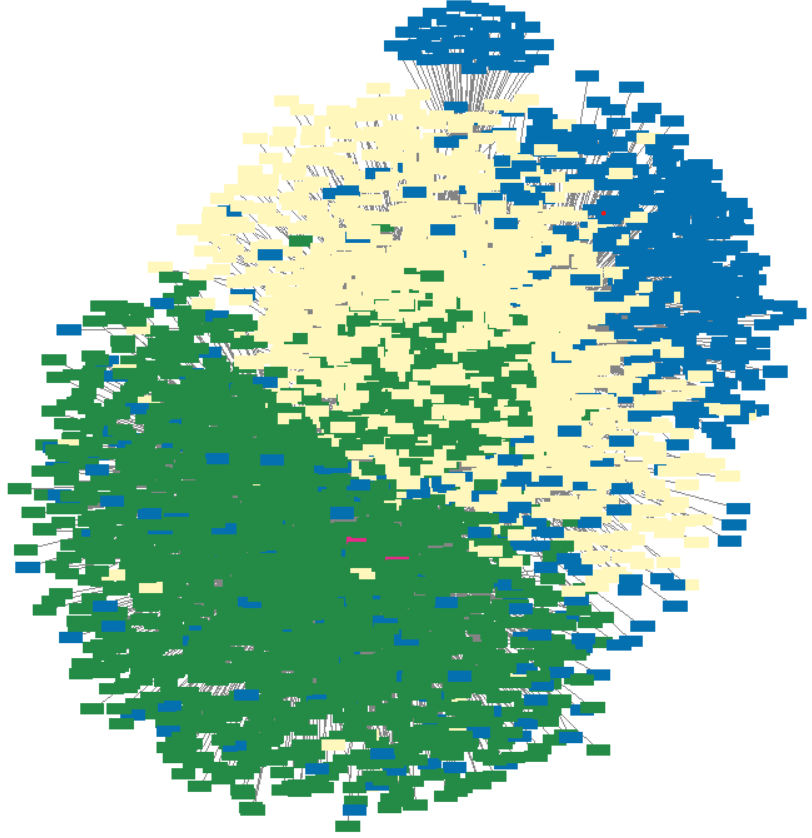
SUPPLEMENTARY ARTICLE**

**Fig S1.** The merged network of shelterin proteins-miRNA-lncRNA-small molecules


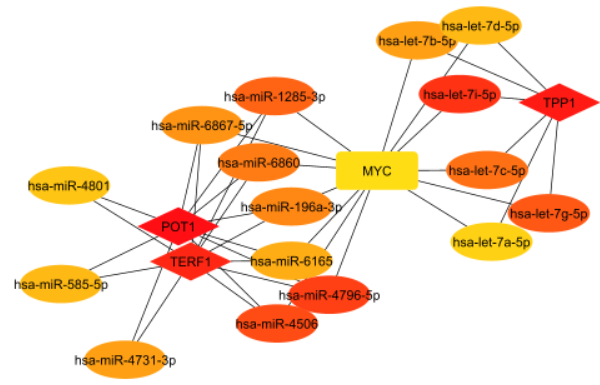


**Fig S2**. The top 20 hub RNA networks from the mRNA-miRNA-TF interaction network


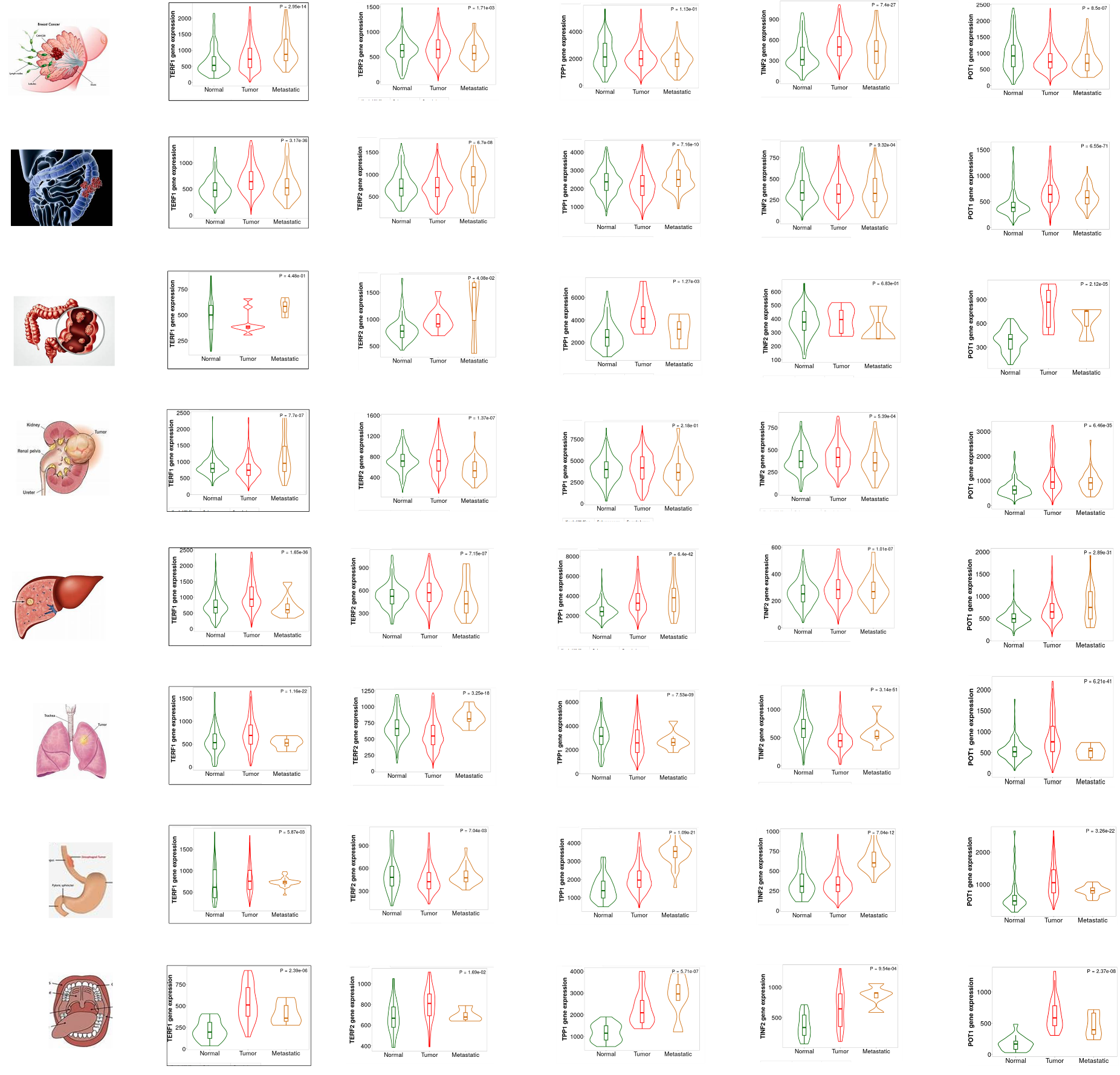


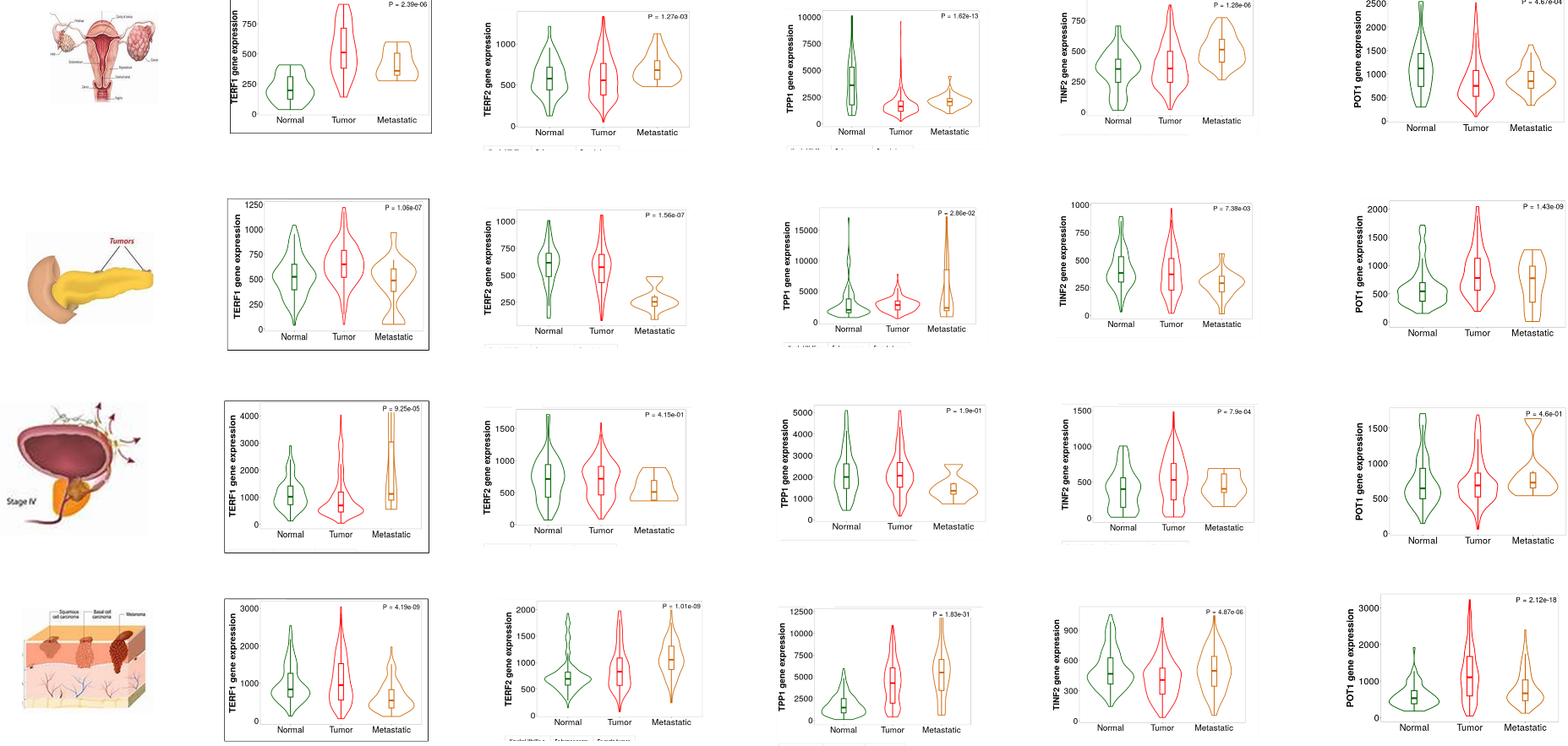


**Fig S2**. The violin plots of the Normal vs. tumour vs. Metastatic expression plots of shelterin proteins


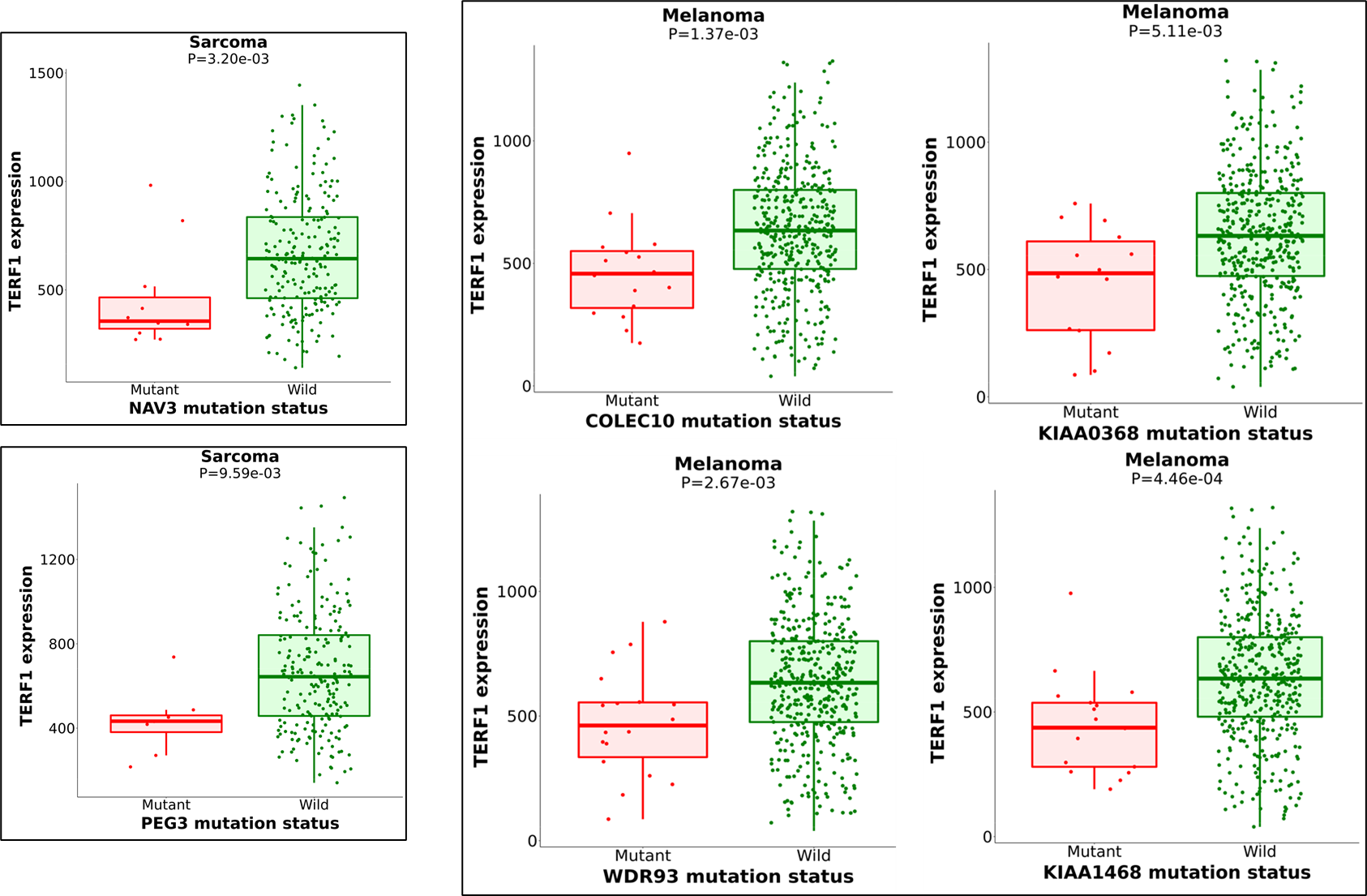

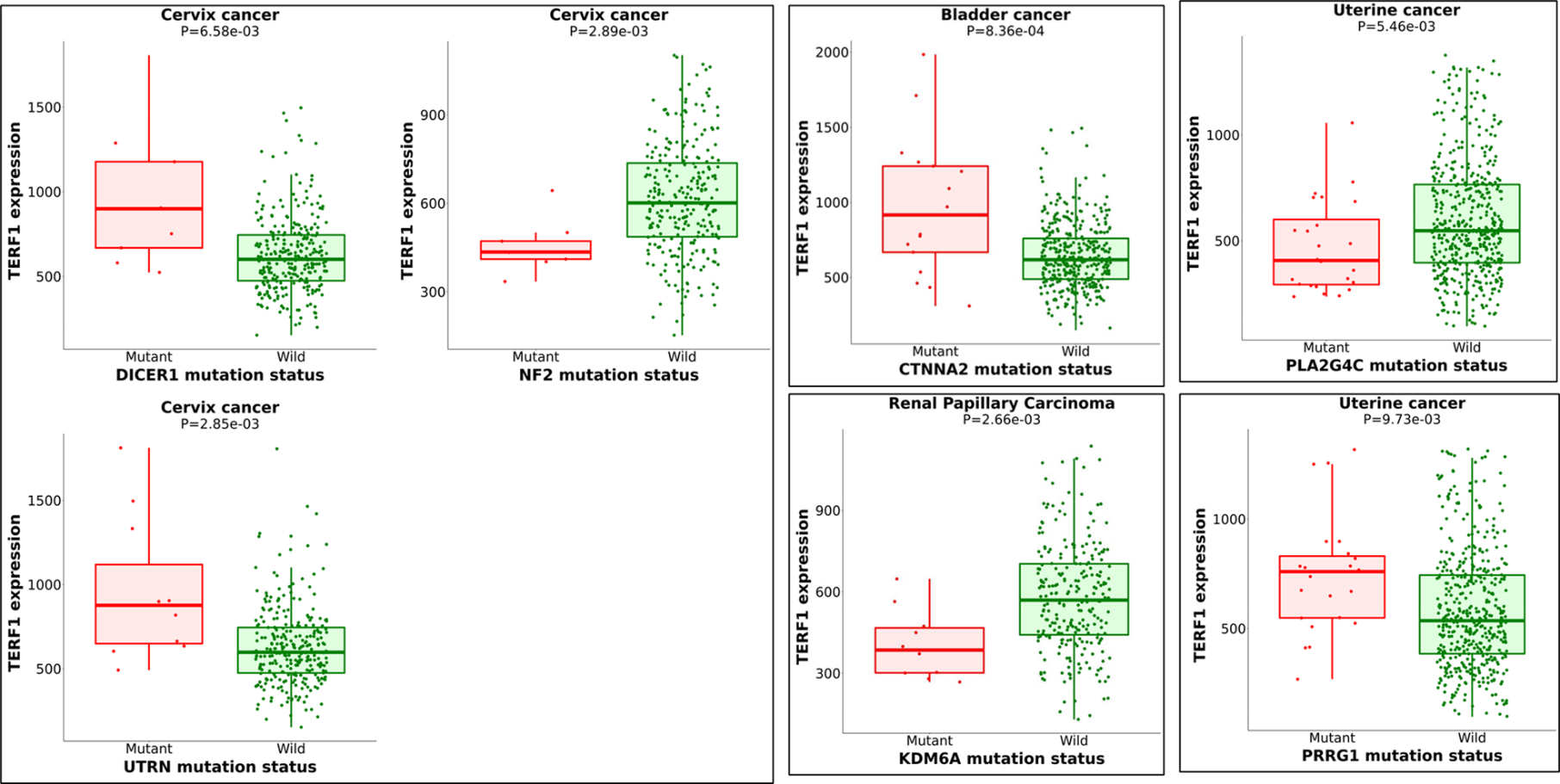


**a**


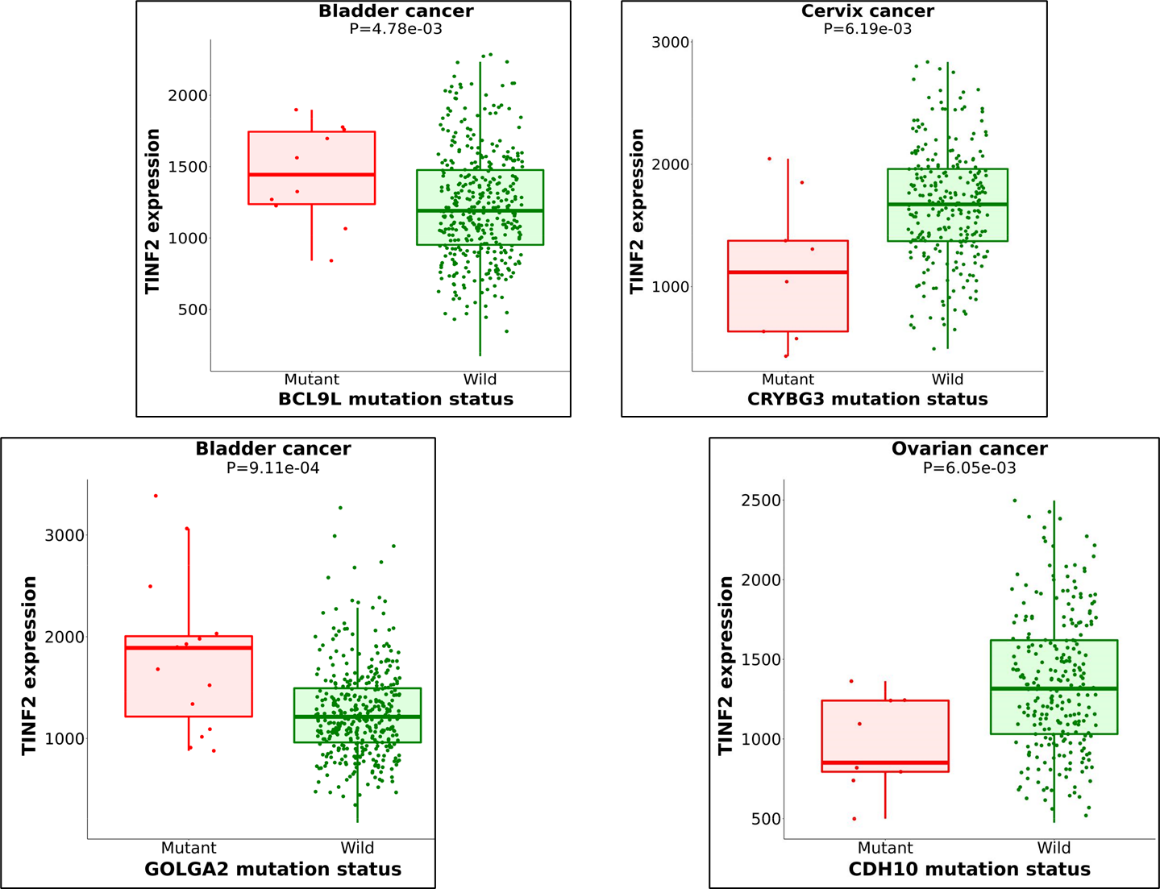


**b**


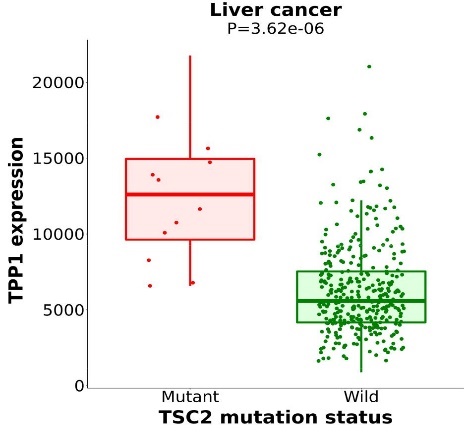

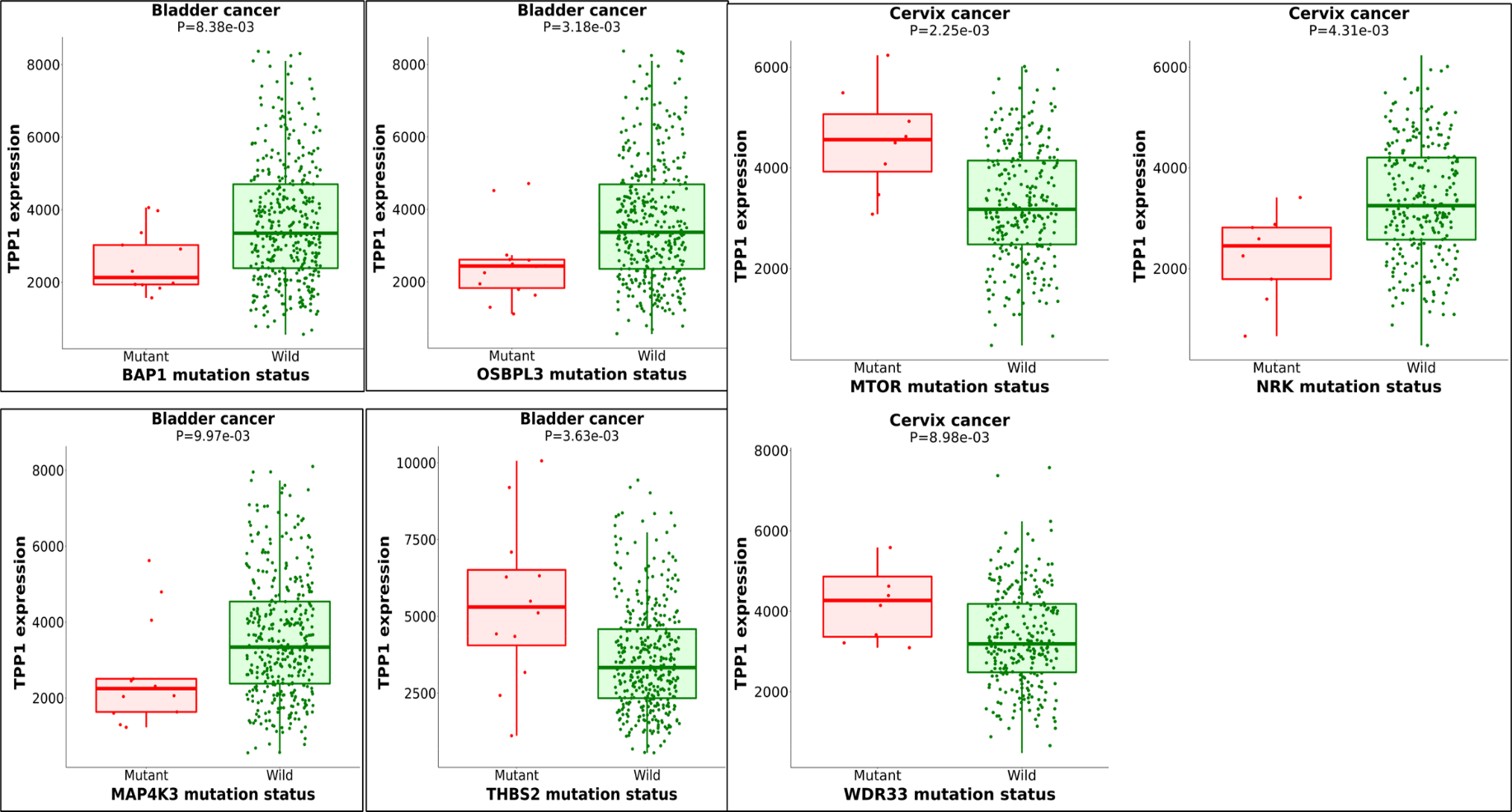


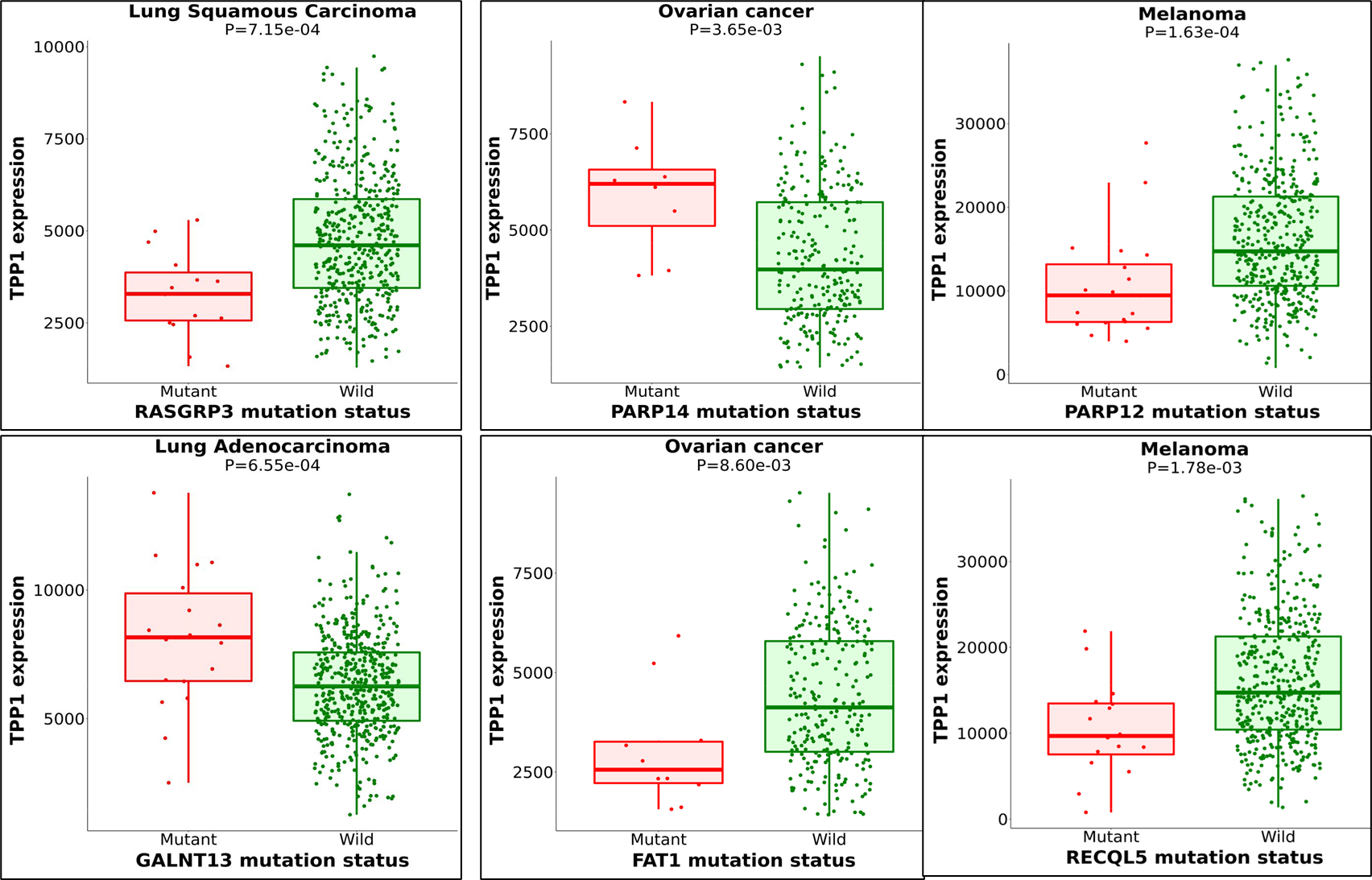

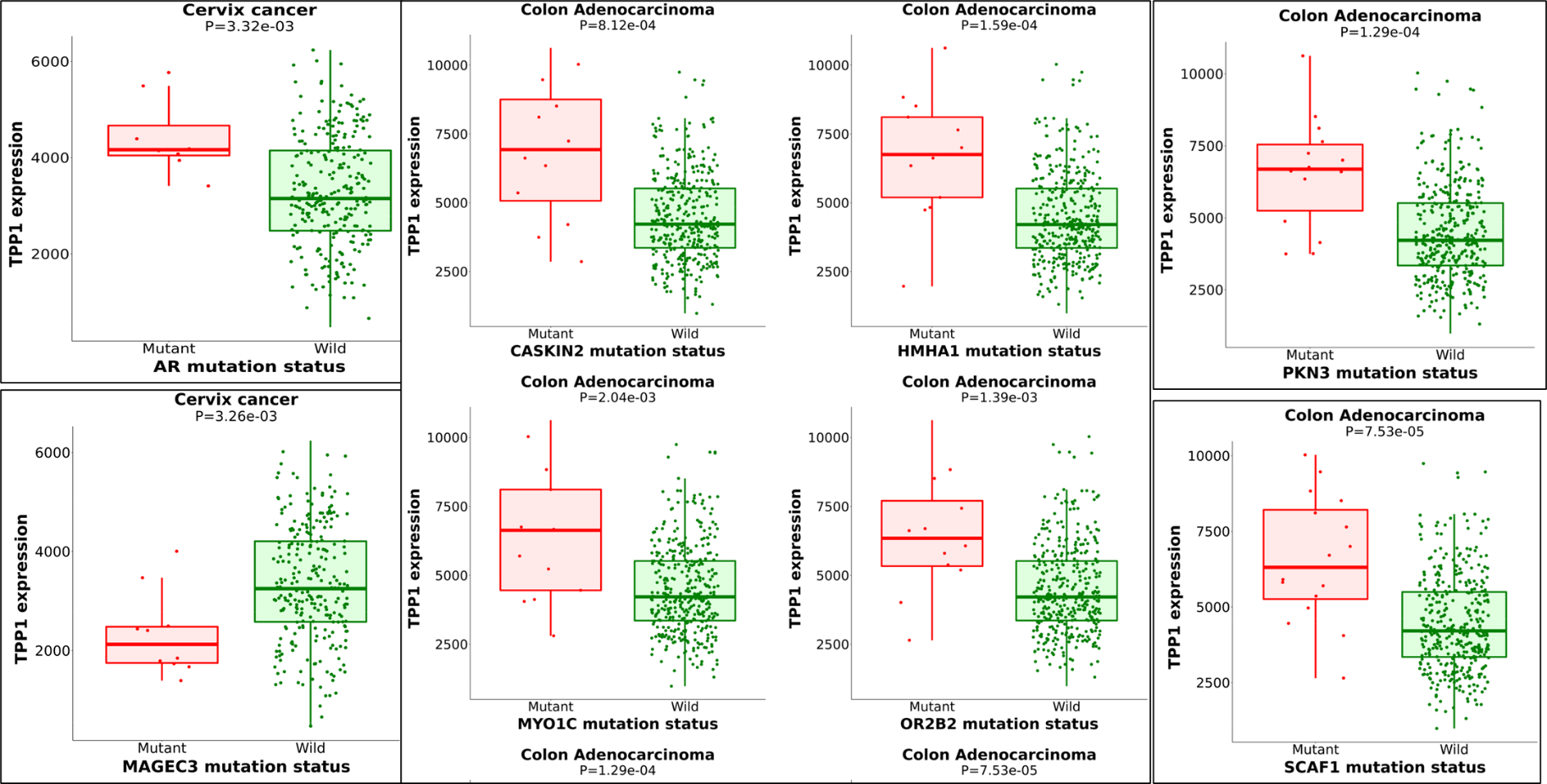


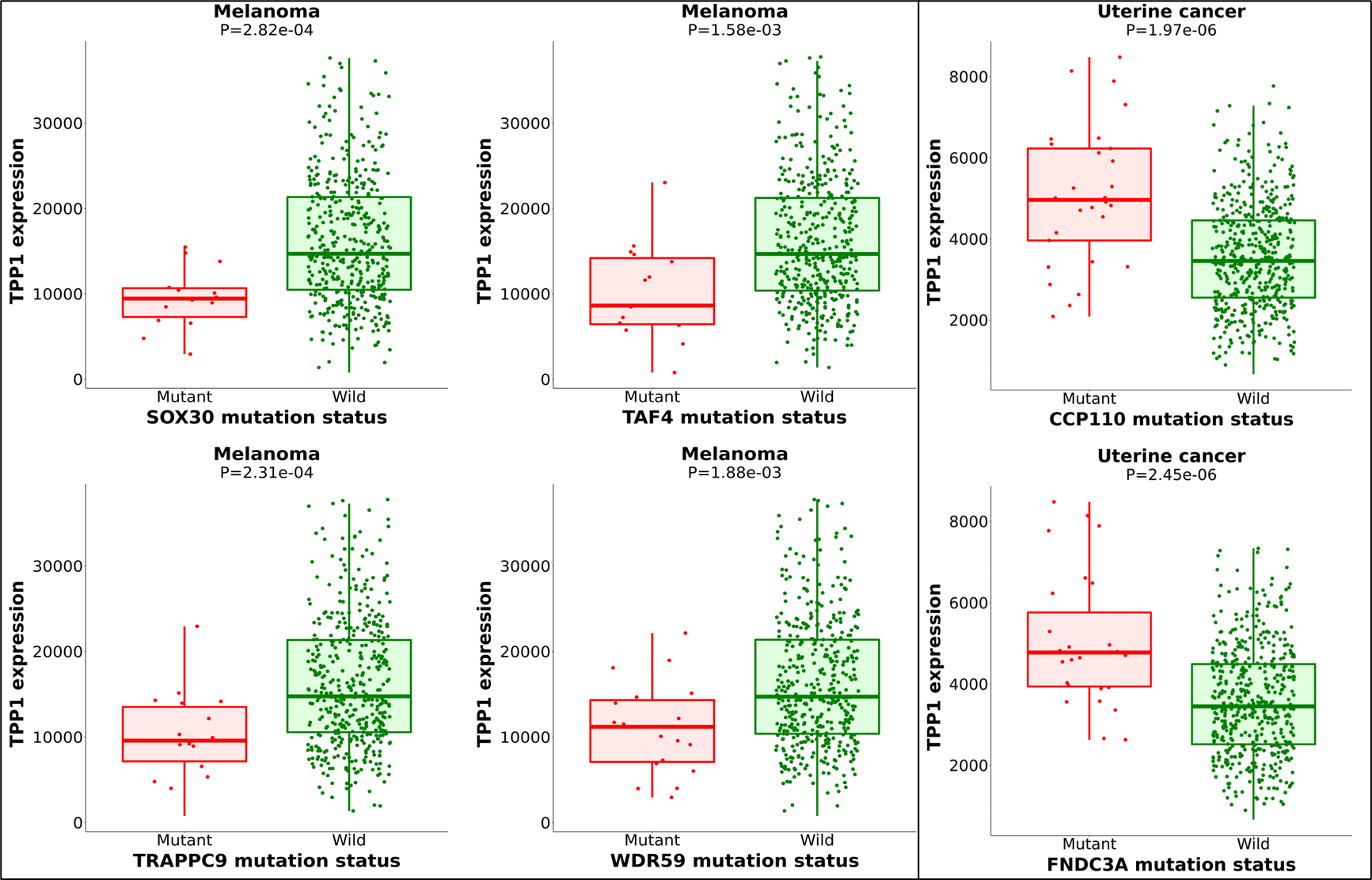

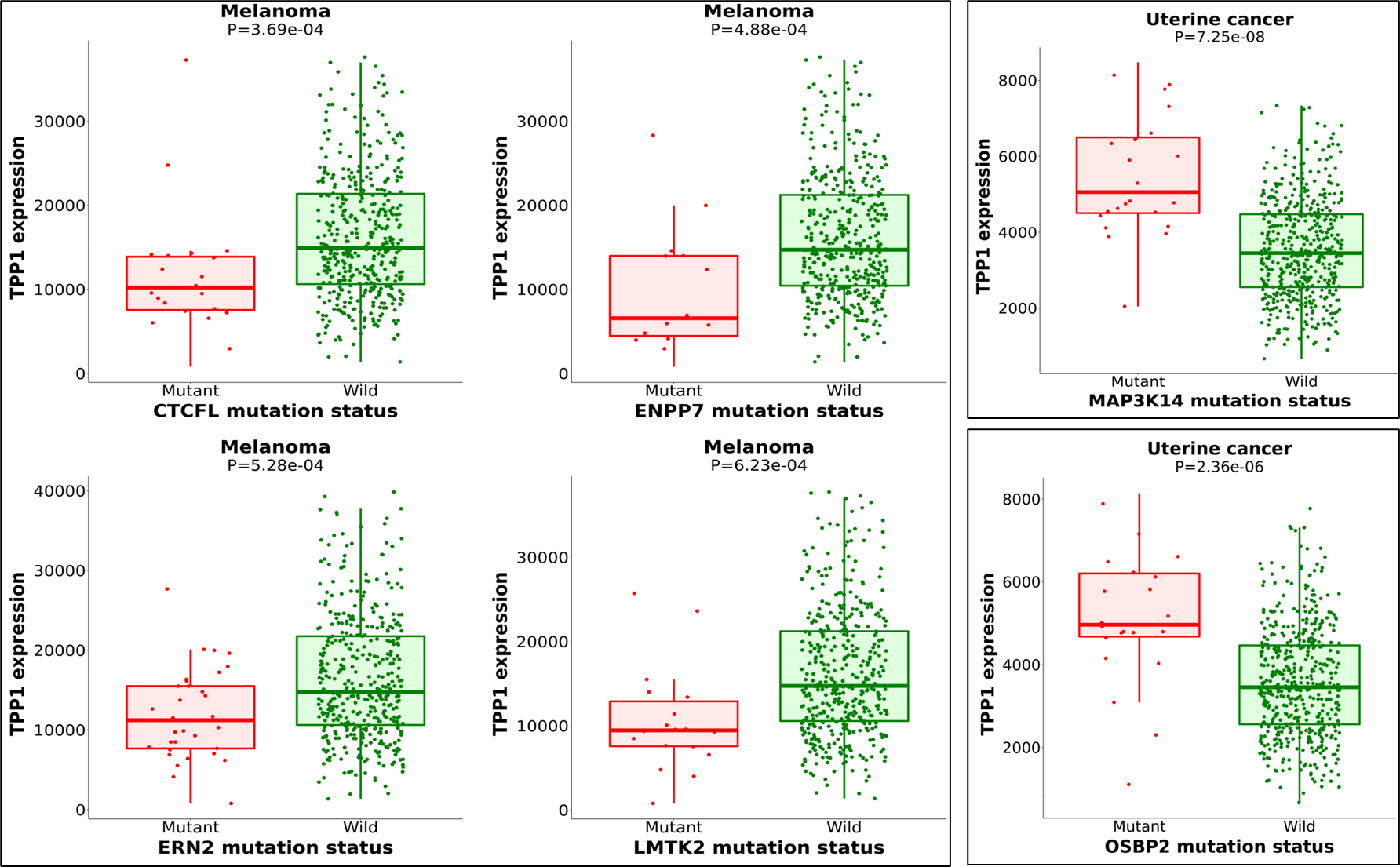


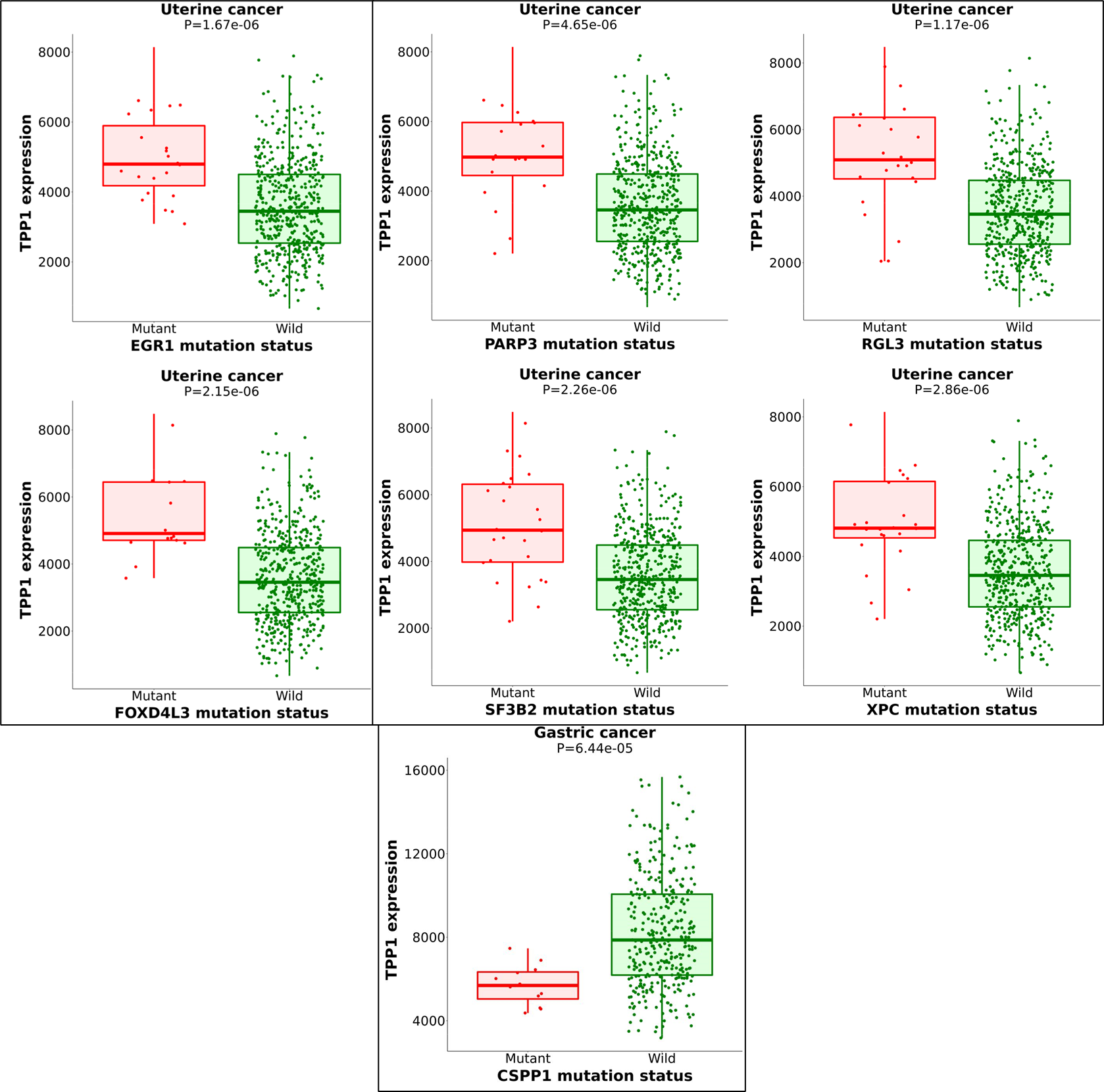


**c**


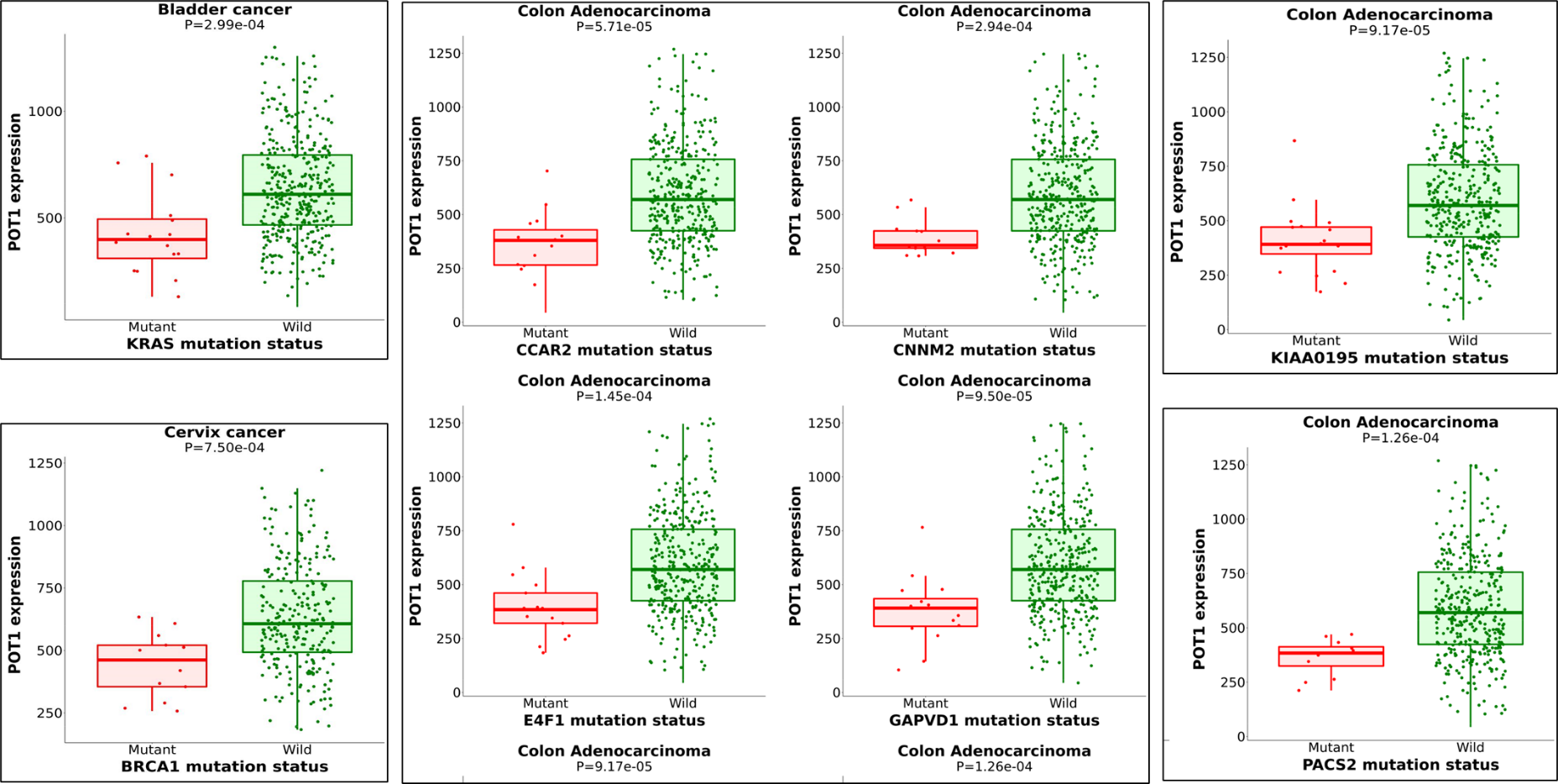

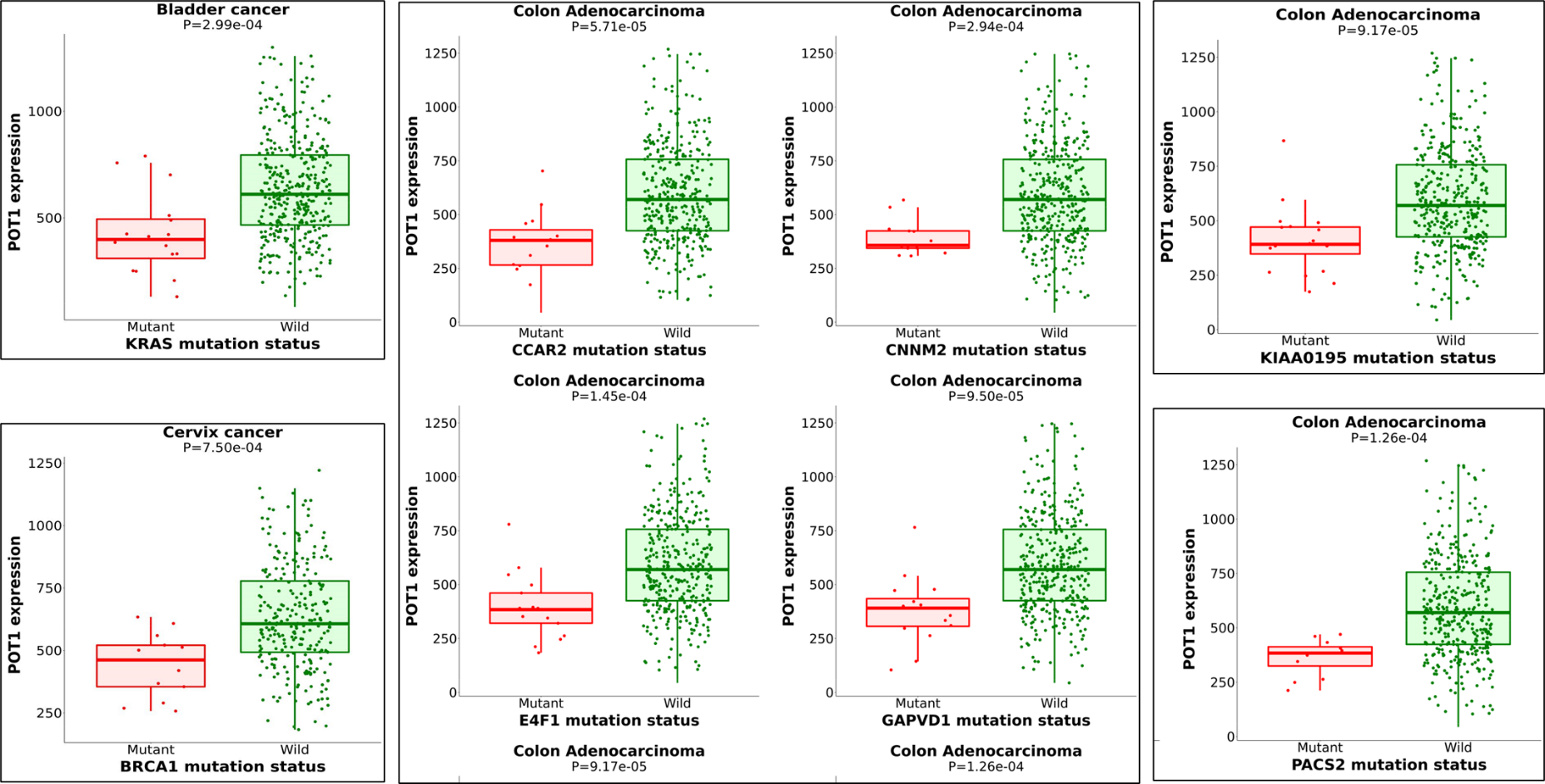


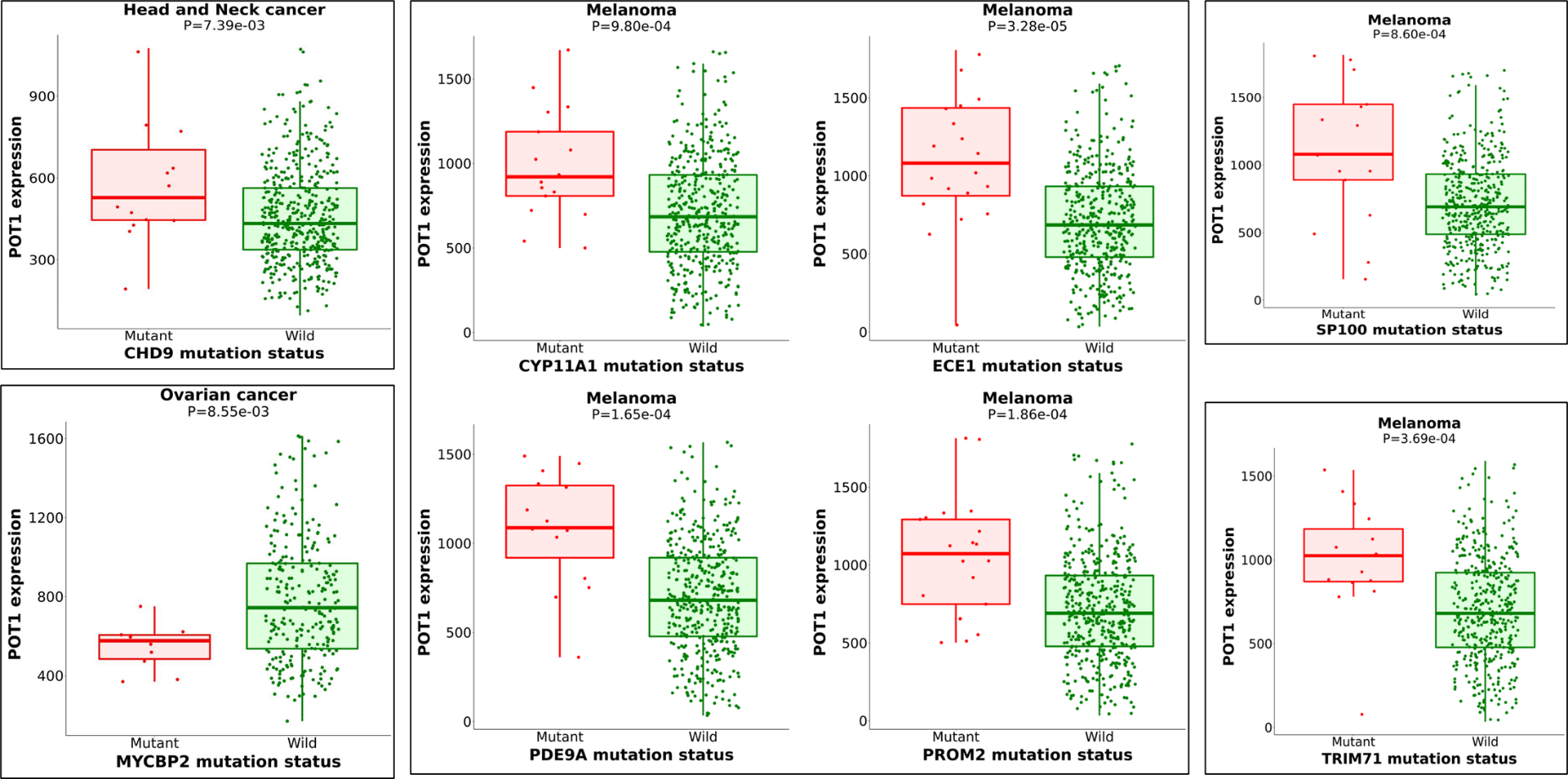

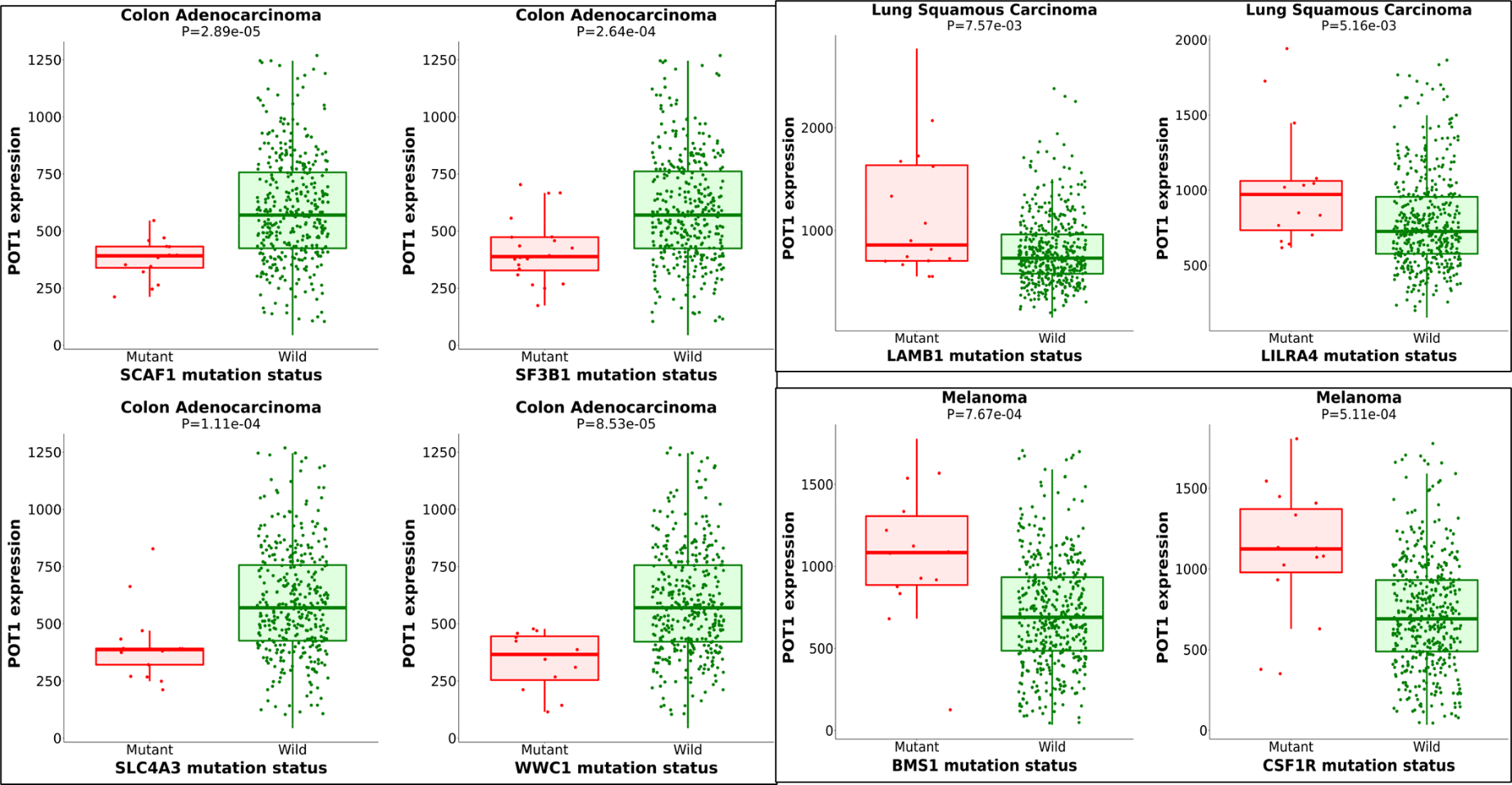


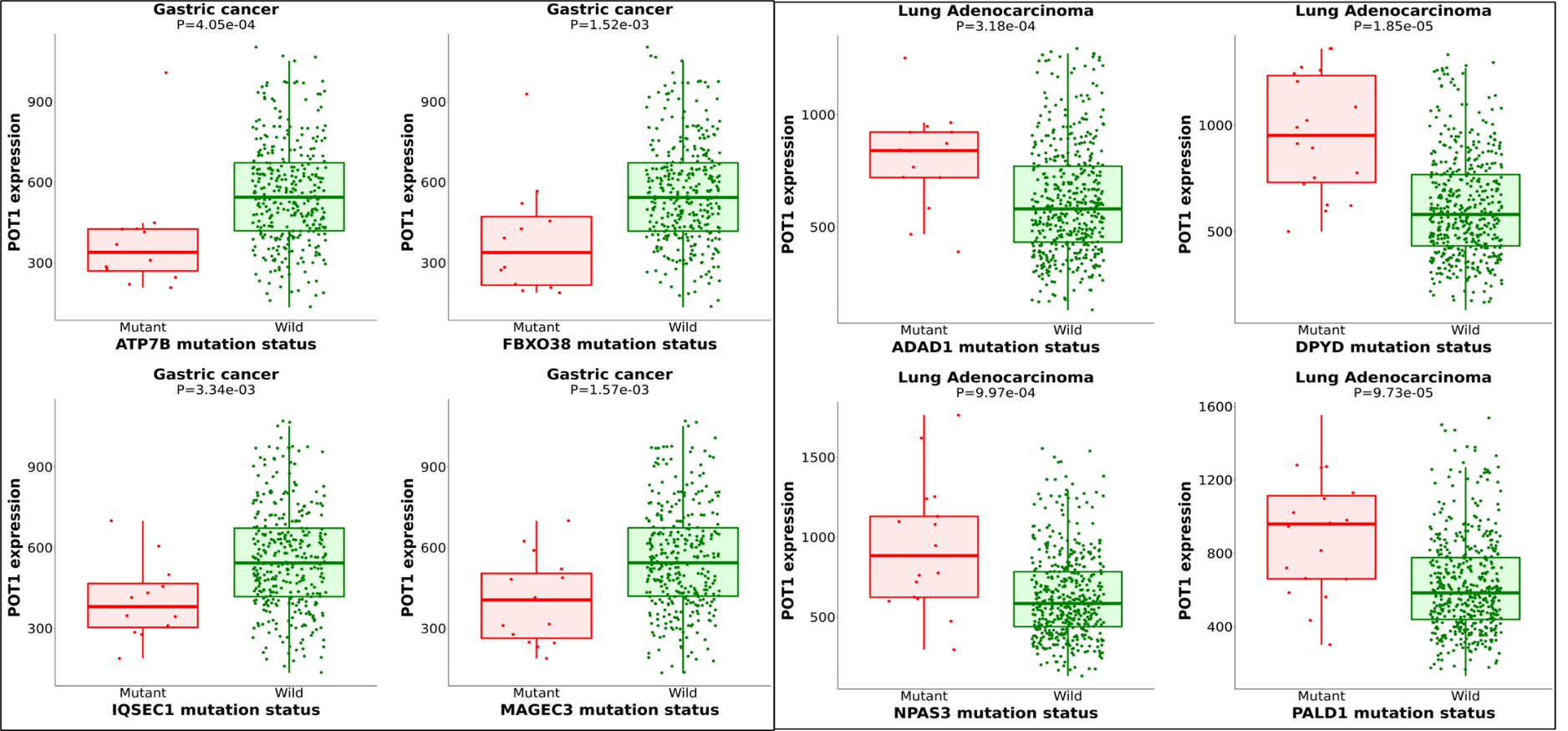

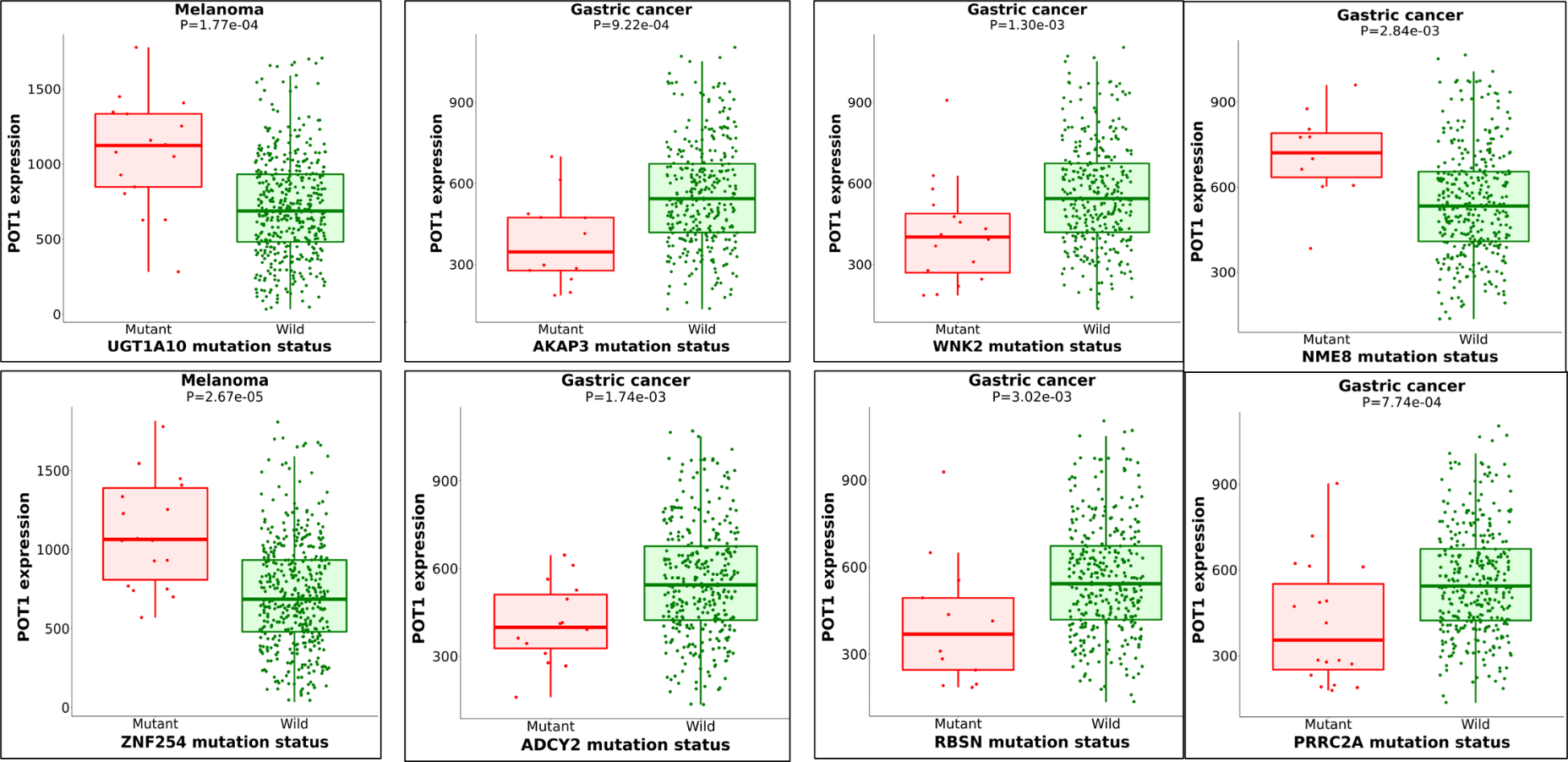


**
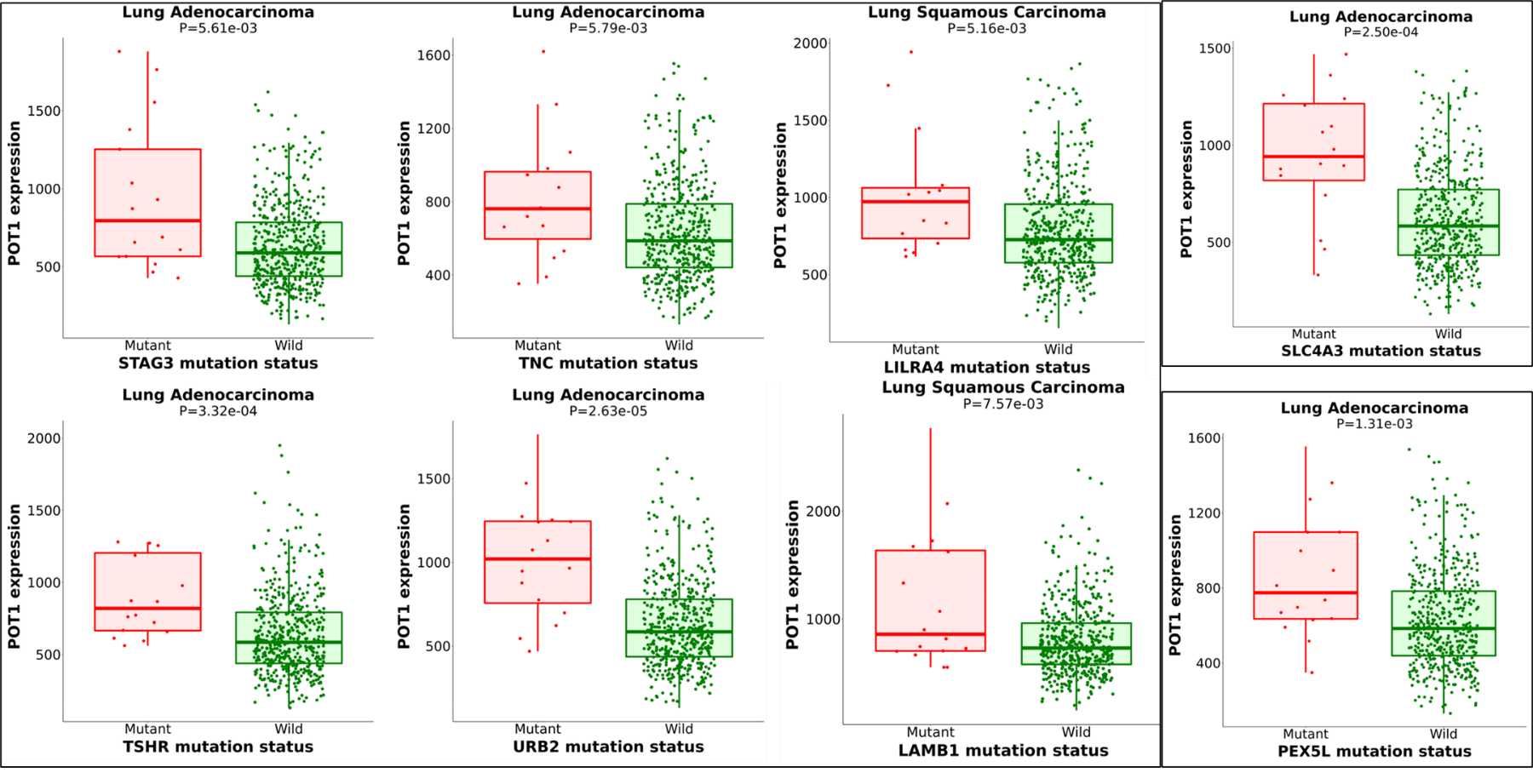
Fig S3**. The mutation status associated with gene expression changes in 18 different solid tumours

**d**

**Table S1.** The list of mutations from cBioPortal respective to the five shelterin proteins

| **Data** | **FD** | **FI** | **MS** | **NS** | **SP** | **IF_DEL** |
| --- | --- | --- | --- | --- | --- | --- |
| TERF1_1 | 0 | 0 | 70 | 0 | 0 | 0 |
| TERF1_2 | 0 | 0 | 17 | 0 | 0 | 0 |
| TERF1_3 | 0 | 0 | 13 | 0 | 0 | 0 |
| TERF1_4 | 0 | 0 | 36 | 0 | 0 | 0 |
| TERF1_5 | 0 | 0 | 334 | 0 | 0 | 0 |
| TERF1_6 | 0 | 0 | 224 | 0 | 0 | 0 |
| TERF1_7 | 0 | 0 | 291 | 0 | 0 | 0 |
| TERF2_1 | 89 | 0 | 0 | 0 | 0 | 0 |
| TERF2_2 | 0 | 0 | 59 | 0 | 0 | 0 |
| TERF2_3 | 61 | 0 | 0 | 0 | 0 | 0 |
| TERF2_4 | 0 | 0 | 21 | 0 | 0 | 0 |
| TERF2_5 | 0 | 0 | 150 | 0 | 0 | 0 |
| TERF2_6 | 0 | 0 | 273 | 0 | 0 | 0 |
| TERF2_7 | 87 | 0 | 0 | 0 | 0 | 0 |
| TERF2_8 | 0 | 0 | 0 | 391 | 0 | 0 |
| TERF2_9 | 0 | 0 | 0 | 0 | 316 | 0 |
| TERF2_10 | 0 | 0 | 0 | 446 | 0 | 0 |
| TPP1_1 | 0 | 0 | 543 | 0 | 0 | 0 |
| TPP1_2 | 0 | 0 | 402 | 0 | 0 | 0 |
| TPP1_3 | 0 | 0 | 216 | 0 | 0 | 0 |
| TPP1_4 | 0 | 0 | 0 | 0 | 6 | 0 |
| TPP1_5 | 0 | 0 | 0 | 242 | 0 | 0 |
| TPP1_6 | 0 | 0 | 208 | 0 | 0 | 0 |
| TPP1_7 | 0 | 0 | 471 | 0 | 0 | 0 |
| TPP1_8 | 166 | 0 | 0 | 0 | 0 | 0 |
| TPP1_9 | 0 | 0 | 260 | 0 | 0 | 0 |
| TPP1_10 | 0 | 0 | 204 | 0 | 0 | 0 |
| TPP1_11 | 0 | 0 | 336 | 0 | 0 | 0 |
| TPP1_12 | 0 | 0 | 0 | 336 | 0 | 0 |
| TPP1_13 | 0 | 0 | 327 | 0 | 0 | 0 |
| TPP1_14 | 0 | 0 | 298 | 0 | 0 | 0 |
| TPP1_15 | 0 | 0 | 251 | 0 | 0 | 0 |
| TPP1_16 | 0 | 0 | 211 | 0 | 0 | 0 |
| TINF2_1 | 0 | 0 | 37 | 0 | 0 | 0 |
| TINF2_2 | 0 | 0 | 265 | 0 | 0 | 0 |
| TINF2_3 | 0 | 0 | 414 | 0 | 0 | 0 |
| TINF2_4 | 0 | 0 | 0 | 221 | 0 | 0 |
| TINF2_5 | 0 | 0 | 421 | 0 | 0 | 0 |
| TINF2_6 | 0 | 0 | 310 | 0 | 0 | 0 |
| TINF2_7 | 0 | 0 | 371 | 0 | 0 | 0 |
| TINF2_8 | 0 | 0 | 371 | 0 | 0 | 0 |
| TINF2_9 | 0 | 0 | 354 | 0 | 0 | 0 |
| TINF2_10 | 0 | 0 | 0 | 423 | 0 | 0 |
| TINF2_11 | 0 | 0 | 0 | 393 | 0 | 0 |
| TINF2_12 | 0 | 0 | 0 | 0 | 171 | 0 |
| TINF2_13 | 0 | 0 | 0 | 0 | 0 | 369 |
| TINF2_14 | 0 | 0 | 0 | 426 | 0 | 0 |
| TINF2_15 | 0 | 0 | 0 | 270 | 0 | 0 |
| TINF2_16 | 0 | 0 | 0 | 241 | 0 | 0 |
| TINF2_17 | 0 | 0 | 0 | 220 | 0 | 0 |
| TINF2_18 | 0 | 0 | 0 | 353 | 0 | 0 |
| TINF2_19 | 0 | 0 | 0 | 400 | 0 | 0 |
| TINF2_20 | 0 | 0 | 0 | 221 | 0 | 0 |
| TINF2_21 | 0 | 0 | 0 | 244 | 0 | 0 |
| POT1_1 | 0 | 0 | 0 | 194 | 0 | 0 |
| POT1_2 | 0 | 0 | 0 | 0 | 502 | 0 |
| POT1_3 | 0 | 0 | 0 | 0 | 42 | 0 |
| POT1_4 | 0 | 0 | 223 | 0 | 0 | 0 |
| POT1_5 | 0 | 0 | 0 | 0 | 13 | 0 |
| POT1_6 | 0 | 0 | 0 | 66 | 0 | 0 |
| POT1_7 | 0 | 0 | 0 | 0 | 182 | 0 |
| POT1_8 | 0 | 50 | 0 | 0 | 0 | 0 |
| POT1_9 | 81 | 0 | 0 | 0 | 0 | 0 |
| POT1_10 | 0 | 0 | 105 | 0 | 0 | 0 |
| POT1_11 | 0 | 0 | 72 | 0 | 0 | 0 |
| POT1_12 | 0 | 0 | 163 | 0 | 0 | 0 |
| POT1_13 | 0 | 0 | 430 | 0 | 0 | 0 |
| POT1_14 | 0 | 0 | 615 | 0 | 0 | 0 |
| POT1_15 | 0 | 0 | 577 | 0 | 0 | 0 |
| POT1_16 | 0 | 0 | 41 | 0 | 0 | 0 |
| POT1_17 | 0 | 0 | 537 | 0 | 0 | 0 |
| POT1_18 | 0 | 0 | 433 | 0 | 0 | 0 |
| POT1_19 | 0 | 0 | 434 | 0 | 0 | 0 |
| POT1_20 | 0 | 0 | 459 | 0 | 0 | 0 |
| POT1_21 | 0 | 0 | 19 | 0 | 0 | 0 |
| POT1_22 | 0 | 0 | 119 | 0 | 0 | 0 |
| POT1_23 | 0 | 0 | 584 | 0 | 0 | 0 |
| POT1_24 | 0 | 0 | 386 | 0 | 0 | 0 |
| POT1_25 | 0 | 0 | 274 | 0 | 0 | 0 |

The Genename_1, _2, _3…., _n indicates the number of mutations at different chromosomal positions for an individual shelterin protein.

**Table S2**. Functional enrichment analysis of shelterin proteins cluster identified BPs, MFs, CCs and Reactome pathways using the STRING database

| **GO-term** | **Term ID** | **Description** | **Genes involved** | **FDR** |
| --- | --- | --- | --- | --- |
| **BP** | 1905839 | Negative regulation of telomeric D-loop disassembly | TERF1  &  TERF2 | 0.00021 |
|  | 1905778 | Negative regulation of exonuclease activity |  |  |
|  | 0032214 | Negative regulation of telomere maintenance via semi-conservative replication |  |  |
|  | 0032202 | Telomere assembly | TINF2 & POT1 | 0.00038 |
|  | 0061820 | Telomeric D-loop disassembly | TERF1, TERF2 & POT1 | 2.70e-06 |
| **MF** | 0098505 | G-rich strand telomeric DNA binding | TERF1, TERF2 & POT1 | 5.53e-06 |
|  | 0003691 | Double-stranded telomeric DNA binding | TERF1 & TERF2 | 0.0018 |
|  | 0042162 | Telomeric DNA binding | TERF1, TERF2, TINF2 & POT1 | 3.97e-07 |
| **CC** | 0070187 | Shelterin complex | TERF1, TERF2, TINF2 & POT1 | 5.38e-10 |
| **REACTOME Pathways** | HSA-174430 | Telomere C-strand synthesis initiation | TERF1, TERF2, TINF2 & POT1 | 4.32e-09 |
|  | HSA-174437 | Removal of the Flap Intermediate from the C-strand |  | 5.44e-09 |
|  | HSA-171319 | Telomere Extension By Telomerase |  | 7.97e-09 |
|  | HSA-174411 | Polymerase switching on the C-strand of the telomere |  | 9.95e-09 |
|  | HSA-171306 | Packaging Of Telomere Ends |  | 2.00e-08 |
